# Is there an association between mental health and economic prosperity? A longitudinal ecological study in England, 2011–2019

**DOI:** 10.1136/bmjopen-2025-113549

**Published:** 2026-06-08

**Authors:** Doriane Mignon, Sam Khavandi, Clare Bambra, Matt Sutton, Luke A Munford

**Affiliations:** 1 School of Health Sciences, HOPE, University of Manchester, Manchester, UK; 2 Department of Business Economics, Health and Social Care (DEASS), Competence Centre for Healthcare Practices and Policies, University of Applied Sciences and Arts of Southern Switzerland (SUPSI), Lugano, Switzerland; 3 Department of Epidemiology and Health Systems, Unisanté, University Center for Primary Care and Public Health and University of Lausanne, Lausanne, Switzerland; 4 Population Health Sciences Institute, Faculty of Medical Sciences, Newcastle University, Newcastle upon Tyne, UK

**Keywords:** MENTAL HEALTH, HEALTH ECONOMICS, Longitudinal studies, Observational Study

## Abstract

**Abstract:**

**Objectives:**

To understand the association between population mental health and economic prosperity at the small area level in England and explore regional differences.

**Design:**

A longitudinal small area-level analysis exploring the association between a proxy for population mental health and economic prosperity across 6789 small areas in England from 2011 to 2019 (N×T=61 101). We apply linear regression models with fixed effects at the area level. Mental health in each area is proxied by a standardised index constructed from administrative data on the use of services related to mental health.

**Setting:**

National study of geographical areas in England.

**Participants:**

Small areas of England.

**Outcome:**

Economic prosperity is measured by gross disposable household income (GDHI) per capita at the small area level.

**Results:**

A one SD increase in the index is linked to a 1.9% (95% CI 1.4% to 2.5%) rise in GDHI. The association varies depending on the region, with the strongest association in the North East.

**Conclusions:**

There is evidence of a positive association between proxies for better population mental health and subsequent household income per capita, varying by region. While we cannot infer causality, these findings are consistent with the view that improving mental health may support local economic prosperity.

**STRENGTHS AND LIMITATIONS OF THIS STUDY**

- We used a validated composite mental health index derived from multiple administrative data sources.
- We used data at the small area level which has not previously been combined and performed an analysis over time.
- We controlled for unobserved time invariant characteristics at the small area level but ultimately our study remains observational, and we cannot assert causality.

## Introduction

 The link between health and economic prosperity has been documented in several respects with early studies showing a positive empirical association between health and economic prosperity, both at the country and individual levels.^1^,^3^ At an aggregated macro level, the association is usually considered in the direction of how economic growth contributes to population health. In their review, Bellés-Obrero and Castelló^4^ focus on the association between business cycle conditions and mortality rates. In many countries, mortality appears to be procyclical although studies on more recent recessions challenge this relationship. Findings regarding mechanisms—such as associations with health behaviours, for example, smoking, drinking, physical activity—are mixed, except for mental health, which consistently deteriorates during recessions. Still at an aggregate level, the impact of health on growth is consistently positive with significant heterogeneity in the magnitude of the effect (see Fumagalli *et al*^5^ for a review). This body of literature primarily relies on cross-country data and typically uses life expectancy as a measure of health.

Evidence on the association between mental health and economic prosperity remains limited. In light of the COVID-19 pandemic and the subsequent rise in the prevalence—and inequality—of mental health issues, the importance of mental health has become even more evident.^6^ The association between individual well-being—and by extension mental health—and economic performance has been extensively studied at the individual level (see the review of Bellet *et al*^7^). However, understanding of this relationship at the aggregate level remains limited. Population mental health may influence area-level economic prosperity through its impact on the labour force. Mental ill-health can reduce individuals’ ability to work and exclude them from the labour market, thereby affecting local economic performance. What has been documented—using both aggregate and micro-level data—is the association between economic growth and mental health, with deteriorations in mental health often linked to recessions,^8^,^11^ although Tekin *et al*,^12^ using microdata, find no significant association (the authors qualify their results as imprecise which they link to potential measurement problems).

The literature studying the association between mental health and economic prosperity at subnational geographical level (eg, regional or local) is more limited, mainly due to data constraints. This paper aims to fill the evidence gap on the association between a proxy for population mental health and future economic prosperity at the area level. The wider literature highlights a bidirectional relationship between economic conditions and mental health: economic circumstances can shape subsequent mental health outcomes, while mental health itself can influence economic prosperity. Adverse economic conditions can worsen mental health through income loss, unemployment and insecurity, while mental ill-health can reduce labour market participation and productivity, with area-level consequences for household income. In this study, however, we focus on one temporal sequence only—the association between population mental health and subsequent area-level economic prosperity. The analysis is therefore associational rather than causal. Our objective is to describe these temporal associations at a fine spatial scale, rather than to estimate causal impacts. While the direction of causality between mental health and economic outcomes has been examined extensively at national and individual levels, there is limited evidence on how such dynamics operate across small geographical areas. Studying the association between health and economic prosperity at a small area level has several strengths for this study. The granularity of the data allows to understand the influence of fine-grained environmental and socioeconomic factors. Particularly relevant to our case, it allows to detect local health inequalities that larger geographies obscure and provide emerging evidence for designing and targeting interventions with much greater precision. By using annual geographical data at Middle-layer Super Output Area (MSOA)-level data over 9 years, we contribute new empirical evidence on temporal associations across local areas in England, while recognising that the data do not support causal inference.

## Methods

### Data

We constructed an annual panel dataset at the MSOA level over the period 2011–2019. MSOA is a statistical geographical level defined by the Office for National Statistics (ONS) containing between 2000 and 6000 households. Data are publicly available and sourced from the ONS website, the Place-Based Longitudinal Data Resource^13^ and Annual Population Survey (APS). The time period was defined by the availability of the main variable of interest, as well as confounding effects associated with the COVID-19 pandemic. Following common practice in small-area research, we excluded MSOAs in the City of London and the Isles of Scilly because of extreme values of the outcome, reflecting statistical instability. More precisely, the City of London is excluded because of extreme outcome values (4.6 times larger than the average of the sample) and the Isles of Scilly because of small population size, which is below the threshold for a MSOA. We therefore have data on 6789 MSOAs which have on average a population of 8062 inhabitants. These are distributed in 307 local authority districts (LAD) and in all nine regions. More details about the different English geographical levels are in online supplemental appendix B.

### Outcome

The main outcome is gross disposable household income (GDHI) per capita measured in 2022 prices. GDHI is the net amount of money that households have available, that is, what they can spend or save, minus the taxes and plus any benefits received. GDHI gives insight into economic activity in the household sector. To be able to compare the values between MSOAs of different sizes, we divide the GDHI by the population of the area. More information about the variable and its construction is available in online supplemental appendix C.1. To account for the fact that pounds in different years are not directly comparable due to differences in prices, we remove the effect of inflation by applying the gross domestic product implicit price deflator to GDHI. We work with GDHI per capita measured in 2022 prices, also called deflated GDHI per capita in the remainder of the text. We log-transformed the variable prior to modelling to interpret the coefficients as percentage changes. The final outcome is the log GDHI deflated per capita. The distributions are displayed in online supplemental appendix (Section A).

### Main variable of interest

Our main predictor is the Small Area Mental Health Index (SAMHI). The index has been constructed based on mental-health related hospital attendances, prescription of antidepressants, depression prevalence and number of recipients of incapacity benefit and employment support allowance for mental illness (see online supplemental appendix C.2 for details about the components and the construction of the variable). SAMHI is a composite indicator intended to capture area-level mental health through multiple administrative measures related to service use, prescribing and disability claims. It therefore reflects both underlying mental health burden and patterns of service utilisation, which are themselves shaped by service availability, clinical practice and policy context. This index ‘is proportional to the overall burden on the healthcare’^14^ but is not an indicator of the prevalence of population mental health. The SAMHI has been validated through use in other ecological studies.^14 15^ We reversed the score (by multiplying the score by −1) to ease the interpretation such that a higher score proxies better area-level mental health, through less service utilisation for mental ill-health-related reasons. The score is normalised so that a one-unit increase represents a one SD increase.

### Controls

Our control variables capture the number of potential workers (aged from 15–65 years old) within the area as well as the age structure, defined as the number of individuals being 15–19, 20–24, 25–29, 30–49, 50–59, 60–64, 65–74, 75–84 and more than 85 years old. We further controlled for the percentage of individuals without any qualifications as a proxy for education levels. We also controlled for the unemployment rate to capture the constraints on employment in the area. These two variables were extracted from the statistics of the APS and are available at the LAD-level.

### Statistical analysis

Our empirical analysis relies on the longitudinal nature of the data, that is, repeated observations of the same MSOA over the period 2011–2019. We ran six linear regression models with different fixed effects specifications to allow progressive comparison of the changes of the main coefficient of interest. Across all models we assumed a log-linear association between the outcome, the log of GDHI per capita for MSOA *i* at time *t*, and the main variable of interest, SAMHI for MSOA *i* at time *t*. We controlled for time *t* with year fixed effects and characteristics time-varying at the MSOA level (population age groups) as well as at the LAD level (unemployment rate and percentage of population with no qualification). Due to missing values for these variables, the dataset is unbalanced. We present the results also for the balanced dataset in online supplemental appendix D.1. We refer to these characteristics as baseline controls when presenting the results. In the first two models we used the contemporary SAMHI value. The first model (model 1) included year fixed effects and baseline controls. It provided us with a baseline of the association between SAMHI and the log of GDHI per capita. This model is likely biased as it does not account for unobservable time invariant characteristics at the area level which are likely to confound the association. In model 2, we added fixed effects at the area (MSOA) level to account for unobservable time invariant characteristics. It also accounts for non-independence of observations because of the repeated measures from the same MSOAs over time. The association is still likely to be biased as it might capture reverse causality, that is, that economic prosperity causes population mental health. As we are interested in the other direction, that is, that population mental health causes economic prosperity, we use the variable SAMHI from the previous time period to introduce a temporal ordering and reduce the concern of reverse causality. It is important to note that including lagged SAMHI values does not identify a causal effect or remove reverse causality. Economic conditions may influence mental health over multiple past periods, and SAMHI itself is relatively persistent over time. The lag specification therefore serves only to examine temporal ordering, not to isolate exogenous variation. As such, the models estimated associations within areas, and the directionality of the relationship cannot be definitively established. We used the SAMHI in the previous time period *t* − 1 in the other models. Model 3 had the same specifications as model 2, except that the main variable of interest is now the SAMHI in the previous time period *t −* 1. We focus on the results for one period lag of SAMHI but we do explore other time lags (−2 and –3), the results are presented in online supplemental appendix D.2.

As we expect MSOAs within an LAD to be affected by similar shocks over time, we show the results for models where we introduced fixed effects interacting times fixed effects with LADs fixed effects to account for unobservable time varying characteristics at the LAD level (model 4). We proceeded similarly for regions (model 5) and Travel To Work Areas (TTWA), which capture labour markets (see online supplemental appendix B) (model 6). This approach allowed us to account for possible different time trends at higher geographical area levels, such as different price variation or specific economic shocks.

To account for the fact that MSOAs are nested within LADs and MSOAs of one LAD are likely to be more alike, we clustered the standard errors at the LAD level.

#### Accounting for limitations of SAMHI

*S*AMHI is a composite indicator of area-level reflecting the use of services (healthcare, prescription, benefits) for mental ill-health-related reasons. The construction of the index relies on factor analysis and the weights attributed to each component indicate how they each contribute to an underlying construct, which is interpreted as population mental ill-health in the area. Because the SAMHI relies on use of service which may vary with service supply, clinical practice and social security policy as well as underlying morbidity, the components can evolve differently over time and by geographical level. We account for this by (1) extensive fixed effects and geography-by-year controls (models 4–6), and (2) sensitivity analyses where we present the results for each component of the SAMHI separately (results in online supplemental appendix D.3). The fixed effects in models 4–6 would control for the possibility that SAMHI is different depending on characteristics which are not varying over time at the geographical level (such as rural/urban) and that the trend in the SAMHI can be different by regions (eg, the level of service use different regionally over time).

### Patient and public involvement

Patients and/or the public were not involved in the design or conduct or reporting or dissemination plans of this research.

## Results

### Mapping regional inequalities

**Figure 1:**
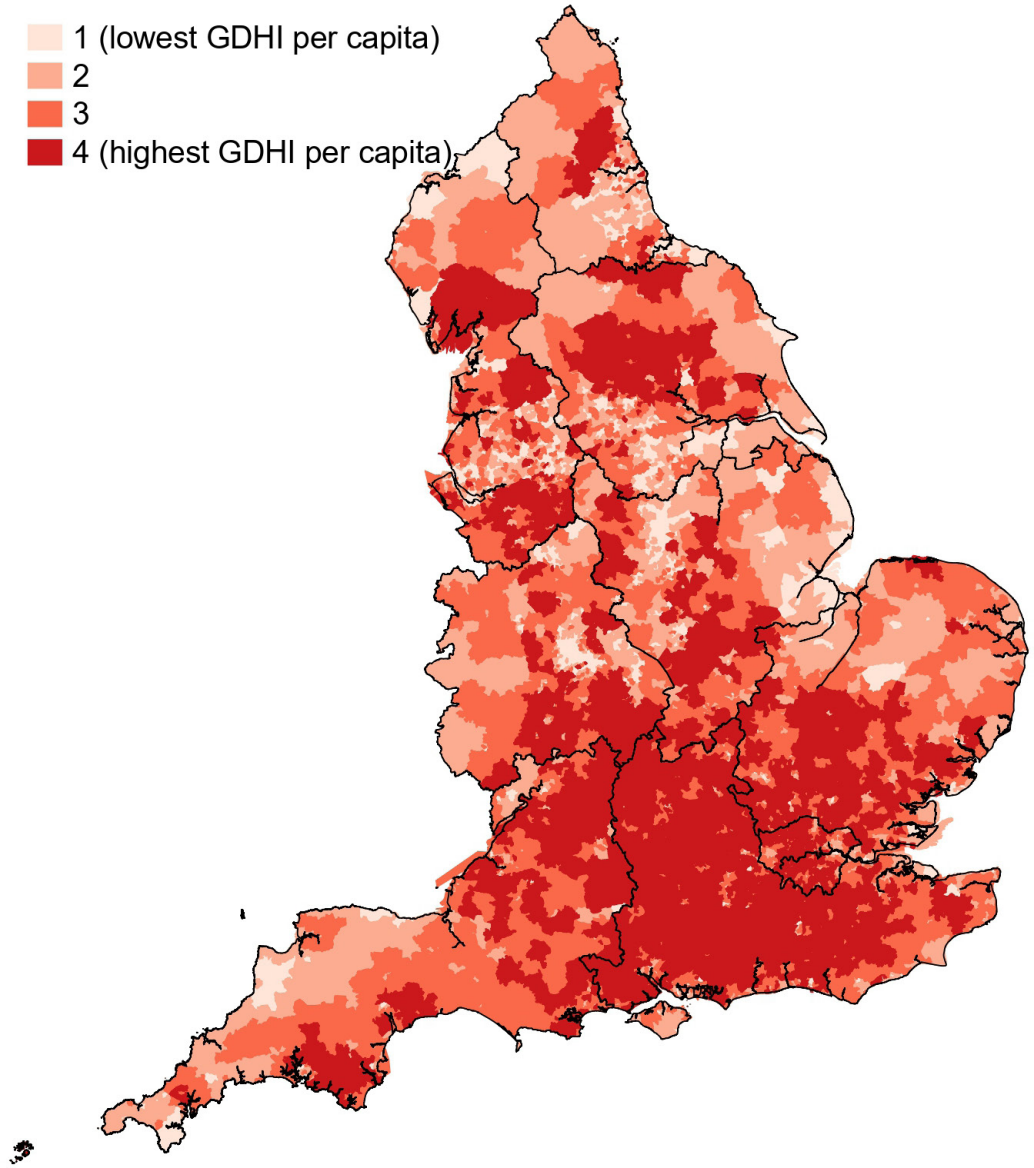
Regional inequalities in economic prosperity—quartiles of GHDI per capita in 2019. The black lines delimit the English regions. The scale represents quartiles with 1 referring to the first quartile and 4 to the last one. GDHI, gross disposable household income.

The mean GDHI per capita over the period is of approximately £22 959 (table 1). There are regional differences in GDHI per capita which have been stable over time (figures1  2). London is at the top of the distribution with the South East in second and the North East at the bottom. This is not surprising as regional inequalities in England have usually been crystallised along the North (North East, North West and Yorkshire and the Humber) versus Rest of England divide. This is also true for the regional inequalities in mental health over time (figure 3). The mean of the SAMHI over the period is 0.02 (table 1), a positive value indicates a score above the period mean. The range of the variable is from –6.01 to 2.07 (not shown in table). The standard deviation (SD) of 0.92 which captures how dispersed the data are from the mean is mainly due to differences between MSOAs (between SD is 0.78; not shown in table), the variation within a MSOA, that is, dispersion in the values for the different observations of one MSOA, exhibits relative persistence of the index (within SD is 0.47; not shown in table). While in 2011 only the North East had a negative value, from 2017, only London’s value is still positive (figure 3). SAMHI has been decreasing over time, signalling more healthcare use for mental health issues and by extension a potential deterioration of mental health.

**Table 1:** Descriptive statistics

Variable	Mean	SD
Outcomes		
GDHI per capita (2022 £)	22 958.81	(6915.58)
Log GDHI per capita (2022 £)	10.01	(0.26)
Mental health variable		
SAMHI	0.02	(0.92)
Control variables available at MSOA level (population by age structure)		
Population aged 15–19	473.48	(207.55)
Population aged 20–24	533.78	(529.63)
Population aged 25–29	560.19	(319.50)
Population aged 30–49	2183.02	(675.74)
Population aged 50–59	1029.79	(247.60)
Population aged 60–64	438.22	(127.85)
Population aged 65–74	755.70	(281.40)
Population aged 75–84	454.11	(180.41)
Population aged 85 and more	187.48	(92.68)
Control variables available at LAD level		
Unemployment rate (%)	6.19	(2.83)
Population without qualifications (%)	8.67	(3.72)

Descriptive statistics over the period 2011–2019 of 6789 MSOAs, excluding City of London and Isles of Scilly.

GDHI, gross disposable household income; LAD, local authority district; MSOAs, Middle-layer Super Output Areas; SAMHI, Small Area Mental Health Index.

**Figure 2:**
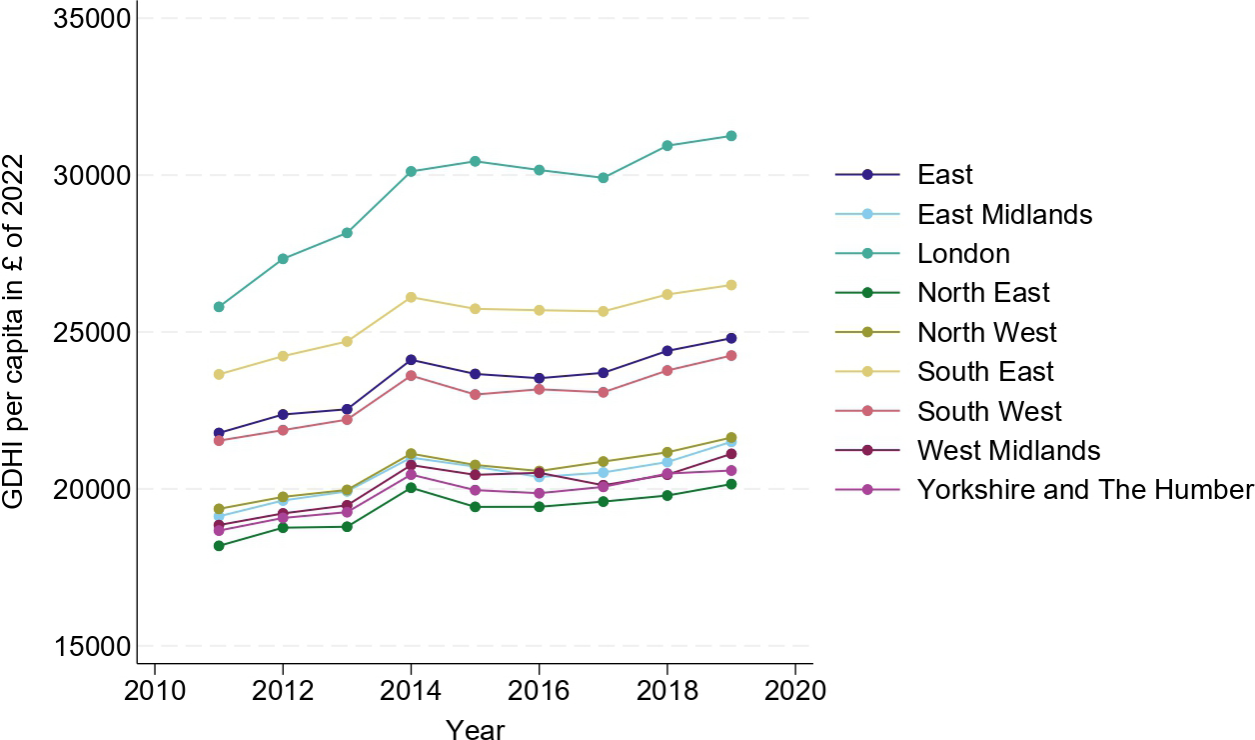
Regional inequalities in economic prosperity—GDHI per capita over time. GDHI, gross disposable household income.

**Figure 3:**
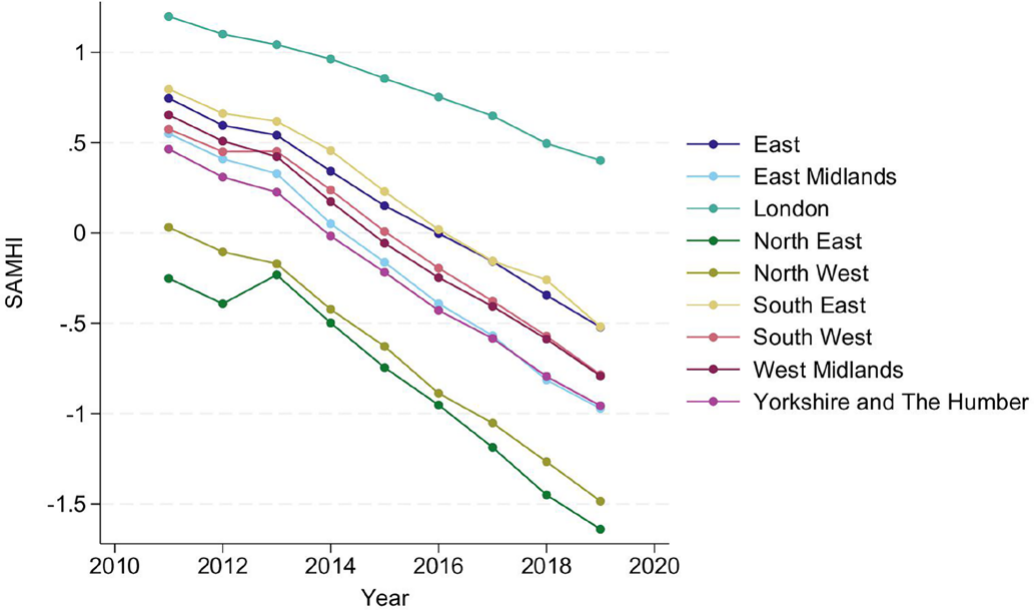
Regional inequalities in proxy of mental health— SAMHI over time. SAMHI, Small Area Mental Health Index.

**Figure 4:**
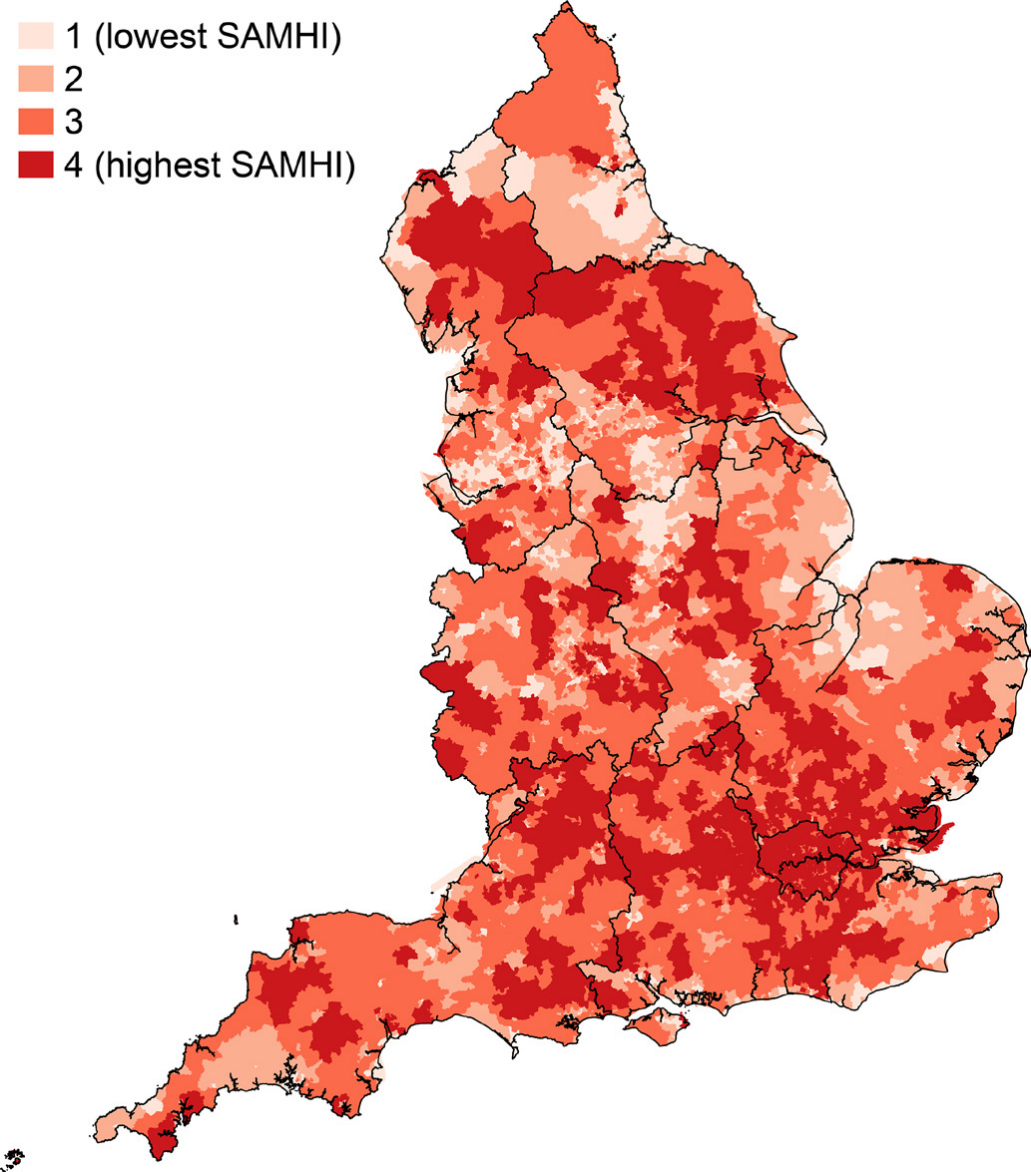
Regional inequalities in proxy of mental health—quartiles of SAMHI in 2019. The black lines delimit the English regions. The scale represents quartiles with 1 referring to the first quartile and 4 to the last one. SAMHI, Small Area Mental Health Index.

Mapping our measures of economic prosperity and the measure of service utilisation related to mental health (figures1  4) showed us that the regions with lower service utilisation and potentially higher underlying mental health seem to be the regions with higher economic prosperity. We also noted that there is a lot of variation within regions with some MSOAs with lowest SAMHI and lowest economic prosperity and at the other extreme MSOAs with highest SAMHI and highest economic prosperity. This is a first indication of an association between economic prosperity and population mental health.

### Association between SAMHI and subsequent GDHI per capita

The association between SAMHI and GDHI per capita is displayed in table 2. Model 1 shows the coefficient of current SAMHI while controlling for population age groups, year fixed effects and controls at the LAD level. We saw a positive and significant association. When introducing MSOA fixed effects in model 2, the coefficient is significantly reduced. When accounting for the possibility of reverse causality and using the lagged value of the SAMHI (model 3), the positive association remained. The coefficient was reduced, which is as expected because reverse causality generates a positive bias. In our preferred specification (model 3, table 2), a one SD increase in SAMHI is associated with a 0.019 (1.9%) increase in GDHI per capita the following year. Accounting for different variation over time in regions (model 4), in labour market assessed by TTWA (model 5) and in LADs (model 6) did not change the results. A one SD increase of the SAMHI is associated with a positive variation of the GDHI per capita, ranging from a 0.006 (0.6%) increase to a 0.012 (1.2%) change depending on the level of time-varying unobservables accounted for.

**Table 2:** Association between log GDHI per capita and SAMHI

	Coeff. (SE)	95% CI	Observations (N×T)	MSOAs (N)
Main variable of interest: SAMHI (SD)				
1. B + Year FE	0.140 (0.007)	(0.125 to 0.154)	57 181	6789
2. B + Year FE + MSOA FE	0.023 (0.003)	(0.017 to 0.028)	57 181	6789
Main variable of interest: lag SAMHI (SD)				
3. B + Year FE + MSOA FE	0.019 (0.003)	(0.014 to 0.025)	50 448	6789
4. B + MSOA FE + Year × Region FE	0.012 (0.002)	(0.007 to 0.016)	50 416	6757
5. B + MSOA FE + Year × TTWA FE	0.009 (0.002)	(0.005 to 0.013)	50 404	6757
6. B + MSOA FE + Year × LAD FE	0.006 (0.002)	(0.002 to 0.010)	50 416	6757

Each line displays the coefficient of one regression. We run six models. The specification of the regressions is indicated at the beginning of the line. Clustered SEs in parentheses at the LAD level. SD is the SAMHI unit. Baseline controls include population age groups, LAD unemployment rate and LAD percentage of population with no qualification. N×T is the number of observations in the regressions, MSOAs (N) the number of MSOAs contributing to the regression.

B, baseline controls ; FE, fixed effects; GDHI, gross disposable household income; LAD, local authority district; MSOA, Middle Super Output Area; SAMHI, Small Area Mental Health Index; TTWA, Travel To Work Area.

### Association by region

**Figure 5:**
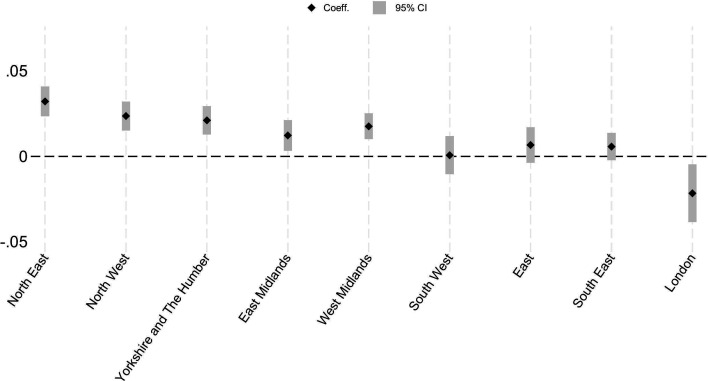
GDHI per capita and SAMHI by regions. A black diamond represents the linear combination of the lag SAMHI coefficient and the interaction term between the lag SAMHI and a region indicator in a regression where the outcome is the log GDHI deflated per capita. The region interacted with is indicated at the bottom. Regions are ordered by ascending mental health average. The grey bar represents the 95% CI. The regression includes as controls population age groups, LAD unemployment rate, LAD percentage of population with no qualification, year and MSOA fixed effects. The SEs are clustered at the LAD level. GDHI, gross disposable household income; SAMHI, Small Area Mental Health Index.

We explored regional inequalities by comparing whether the association is the same across the different regions. We interacted the one-period lag of SAMHI with region indicators and compared the linear combinations of the coefficient of the lag SAMHI and the interaction term (figure 5). The reference region was East so the coefficient related to this region is the coefficient of the lag SAMHI. We saw evidence of significant regional heterogeneity of the association as the CIs of the coefficients do not always overlap. The 0.019 significant coefficient (model 3, table 2) seems to be mainly driven by the regions North East, North West, Yorkshire and The Humber, East Midlands and West Midlands which all have positive and significant coefficients. The highest positive coefficient is for the North East (0.032). Interestingly, the coefficient for London is negative and significant: a positive change of SAMHI is associated with lower GDHI per capita.

## Discussion

### Statement of the principal findings

In this study, we set out to examine the association between mental health and subsequent economic prosperity at the small-area level in England. We use the SAMHI reflecting service use for mental ill-health-related reasons as a proxy for mental health. The longitudinal nature of our data enabled us to introduce MSOA fixed effects in our models (models 2–6), which control for unobservable time-invariant characteristics at the area level. On average, we found a positive association: an increase in SAMHI is associated with higher GDHI per capita the following year. These results remained robust to the inclusion of time-varying characteristics at higher geographic area levels (models 4, 5 and 6).

In our preferred specification, which used the time-lagged value of the SAMHI and included year and MSOA fixed effects (model 3, table 2), a one SD increase in SAMHI is associated with a 1.9% increase in GDHI per capita the following year, which means that a 0.1 SD increase is associated with ~0.19% higher GDHI per capita per year (about £44 at the sample mean), and a 0.2 SD increase with ~0.38% (about £87). GDHI per capita including all residents (including children and inactive adults), this number is an average which applies to every resident of the region, not only the working population. Given the existence of substantial regional inequalities, we explored whether the association of interest varies across regions. The underlying question was whether investments in mental health would yield similar economic returns across all regions. We do find significantly different coefficients (figure 3) with most of them (five) being positive and significant, three being non-significant and even the coefficient for London being negative and significant. Regional heterogeneity may reflect differences in structural characteristics or decreasing returns to scale, given that each region starts from a different baseline level of SAMHI.

### Strengths and weaknesses of the study

Our coefficient estimates should be interpreted with caution. Our empirical analysis allowed us to account for time-invariant characteristics, and even when we controlled for time-varying characteristics at the LAD level by interacting time fixed effects with LAD fixed effects (model 6, table 2)—a positive association between population mental health and GDHI per capita persists. However, we could not entirely rule out the possibility that the significant positive coefficient is due to bidirectional causality and dynamic confounding, that is, that past GDHI per capita affect both SAMHI in the subsequent period and GDHI per capita in the subsequent period. Modelling this dynamic is out of the scope of this study, but it means that our estimates may be biased and probably an upper bound of the true effect of past mental health use on subsequent GDHI per capita. Even if the data are a strength of this study, they have limitations. First, our approach at the area level enabled us to draw conclusions at an aggregated level. However, this ecological approach suffers from potential limits. While the ecological approach adopted means that we are avoiding atomistic fallacy, it does not mean that we can draw conclusions on the association between mental health and economic prosperity at the individual level because we cannot rule out ecological fallacy. Second, we relied on a composite index which might have limitations.^16 17^

SAMHI’s strengths are small-area coverage and multisource design, but three limitations are salient. First, SAMHI is a proxy that integrates service use, prescribing and benefit claiming; each component is sensitive to local availability, clinical practice and eligibility rules, and thus may diverge from true morbidity. Recent work showed indeed that the proportion of people with depression receiving treatment has increased over time.^18^ We therefore avoid framing results as changes in prevalence and instead describe changes in the use of services for mental ill-health-related reasons. Second, the time-varying composition of the index (eg, strong upward trend in antidepressant prescribing; discontinuation/replacement of benefits series after 2019) complicates the attribution of longitudinal patterns to any single driver.^13 19^ Third, geographical heterogeneity—in workforce, access and care-seeking attitudes—means that SAMHI may capture unmet need differently across areas.^20^,^22^ These issues do not invalidate SAMHI’s use, but they constrain causal interpretation; we therefore keep policy claims modest and focus on relative area differences and patterning. While a strength of the SAMHI is to rely on administrative exhaustive data which relates to services use for mental ill health, this is capturing only an aspect of population mental health, as the absence of mental ill health does not equal positive mental health. Due to the lack of data of positive mental well-being at the geographical level of interest (MSOA), we are limited in that regard, and this remains out of the scope of this study.

### Strengths and weaknesses in relation to other studies

We contributed to the literature of the determinants of regional economic prosperity by considering mental health, which had not been studied before, and to the literature studying the interdependencies between mental health and labour market outcomes at an aggregated level. The health of the labour force and how it participates in regional economic prosperity has been studied in Bambra *et al*.^23^ We extend this work by considering population mental health and using data at a smaller geographical area level.

### Meaning of the study and implications for policymakers

Our empirical approach allowed us to uncover a positive association between a proxy for population mental health and economic prosperity. Even if we cannot draw causal conclusions from our results, as the British government continues to promote levelling up policies and has characterised the National Health Service as a vector for economic productivity, it is important to recognise the contribution of mental health. The cost of mental health issues is to be considered not only on the direct cost related to treatment but also on the indirect cost through its effects on the economy.^24^ Policies that promote and improve mental health will improve people’s well-being, but one needs to further explore how to translate this into an increase in economic performance.

## Supplementary material

10.1136/bmjopen-2025-113549 online supplemental file 1

## Data Availability

Data are available upon reasonable request.

## References

[1] Bloom DE, Canning D (2000). The Health and Wealth of Nations. Science.

[2] Bloom DE, Canning D, Sevilla JP (2001). The effect of health on economic growth: theory and evidence.

[3] Suhrcke M, McKee M, Stuckler D (2006). The contribution of health to the economy in the European Union. Public Health.

[4] Bellés-Obrero C, Castelló JV (2018). Oxford research encyclopedia of economics and finance.

[5] Fumagalli E, Pinna Pintor M, Suhrcke M (2024). The impact of health on economic growth: A narrative literature review. Health Policy.

[6] Bambra C, Munford L, Khavandi S (2023). Northern *e*xposure: COVID-19 and *regional inequalities in health and weal*th.

[7] Bellet CS, De Neve J-E, Ward G (2024). Does Employee Happiness Have an Impact on Productivity?. Manage Sci.

[8] Charles KK, Decicca P (2008). Local labor market fluctuations and health: is there a connection and for whom?. J Health Econ.

[9] McInerney M, Mellor JM (2012). Recessions and seniors’ health, health behaviors, and healthcare use: analysis of the Medicare Current Beneficiary Survey. J Health Econ.

[10] Bradford WD, Lastrapes WD (2014). A prescription for unemployment? Recessions and the demand for mental health drugs. Health Econ.

[11] Niedzwiedz CL, Thomson KH, Bambra C (2020). Regional employment and individual worklessness during the Great Recession and the health of the working-age population: Cross-national analysis of 16 European countries. Soc Sci Med.

[12] Tekin E, McClellan C, Minyard KJ (2013). Health and health behaviors during the worst of times.

[13] Daras K, Barr B (2021). Small area mental health index (samhi) version 4.00.

[14] Petersen J, Alexiou A, Brewerton D (2022). Impact of selective licensing schemes for private rental housing on mental health and social outcomes in Greater London, England: a natural experiment study. BMJ Open.

[15] Fahy K, Alexiou A, Daras K (2023). Mental health impact of cuts to local government spending on cultural, environmental and planning services in England: a longitudinal ecological study. BMC Public Health.

[16] Cairns JM, Curtis SE, Bambra C (2012). Defying deprivation: A cross-sectional analysis of area level health resilience in England. Health Place.

[17] Cairns-Nagi JM, Bambra C (2013). Defying the odds: A mixed-methods study of health resilience in deprived areas of England. Soc Sci Med.

[18] Clery E, Morris S, Wilson C, Morris S, Hill S, Brugha T (2025). Adult psychiatric morbidity survey: survey of mental health and wellbeing, England, 2023/4.

[19] Heald AH, Stedman M, Davies M (2020). Influences on the use of antidepressants in primary care: All England general practice‐level analysis of demographic, practice‐level and prescriber factors. Hum Psychopharmacol.

[20] Beazley P (2024). Mental health workforce in England: regional trends and disparities. British J Mental Health Nurs.

[21] Wang RAH, Smittenaar P, Thomas T (2024). Geographical variation in perceptions, attitudes and barriers to mental health care-seeking across the UK: a cross-sectional study. BMJ Open.

[22] Maconick L, Sheridan Rains L, Jones R (2021). Investigating geographical variation in the use of mental health services by area of England: a cross-sectional ecological study. BMC Health Serv Res.

[23] Bambra CL, Munford L, Brown H (2018). Health for wealth: building a healthier northern powerhouse for UK productivity.

[24] Layard R (2013). Mental health: the new frontier for labour economics. IZA J Labor Policy.

