# Supplementary material for: Is there an association between mental health and economic prosperity? A longitudinal ecological study in England, 2011–2019
Source: BMJ Open. 2026 Jun 8;16(6):e113549. doi: 10.1136/bmjopen-2025-113549 (PMC13250192; doi:10.1136/bmjopen-2025-113549)

**Appendix**

# Economic prosperity distribution

Figure A.1: Distribution of outcome variable

(a) Distribution of deflated GDHI per capita (b) Distribution of log deflated GDHI per capita

***[Insert FigureA1_combine here]***

# English geographical levels

| Name | Number |  | Mean population |
| --- | --- | --- | --- |
| Region | 9 |  | 6,794,266 |
| Travel To Work Area (TTWA) | 155 |  | 1,870,128 |
| Local Authority District (LAD) | 309 |  | 264,272 |
| Middle Super Output Area (MSOA) | 6,791 |  | 8,288 |
| Lower Super Output Area (LSOA) | 32,844 |  | 1,713 |

***[Insert FigureB_regions here]***

Region and Local Authority District are administrative levels. Travel To Work Area are empirically defined based on commuting patterns. The lowest level for census statistics is Output Area. Each Output Area is made up of between 40 and 250 households and a usually resident population of between 100 and 625 persons. Lower Super Output Area are made up of groups of Output Areas (OAs), usually four or five. Middle layer Super Output Areas (MSOAs) are made up of groups of Lower layer Super Output Areas (LSOAs), usually four or five (h[ttps://www.ons.gov.uk/census/census2021dictionary/areatypedefinitions](http://www.ons.gov.uk/census/census2021dictionary/areatypedefinitions)).

# Measures details

## Economic prosperity measure - Gross Household Disposable Income (GDHI)

Gross disposable household income (GDHI) is the amount of money that individuals in households have available for spending or saving after they have paid taxes and received benefits. The total amount at the MSOA level is data readily available from the ONS website. More information is available on the ONS website: h[ttps://www.ons.gov.uk/economy/regionalaccounts/grossdispos-](http://www.ons.gov.uk/economy/regionalaccounts/grossdispos-) ablehouseholdincome/articles/disaggregatinguksubnationalgrossdisposablehouseholdincometolowerlevelsofgeography/2002to2021. We divide GDHI of each MSOA by the population in the MSOA in that year. This allows us to compare MSOAs of different sizes.

## Mental health measures

### Mental health related hospital attendances

This indicator is a count of all emergency and elective admissions which related to mental health issues. It encompasses admissions which have codes indicating intentional self-harm, event of undetermined intents, mental and behavioural disorders and alcohol consumption related events. The full list of the diagnosis code is available here

### Antidepressants prescription

The indicator is based on the average daily quantity of antidepressants that are prescribed on a quarterly basis by all practices in England.

### Depression prevalence

Depression prevalence measure comes from the estimated number of patients with a diagnosis of depression reported at the GP practice level. The reporting is done in the context of Quality and Outcomes Framework (QOF), which is a voluntary reward incentive programme for GP practices.

### Incapacity benefit and employment support allowance (IBESA) for mental illness

The indicator is based on the estimated number of people claiming Incapacity benefit and Employment support allowance (IBESA) for mental illness. Department for Work and Pensions (DWP) delivers Incapacity Benefit if individuals are too unwell to work. From the 31^st^ of January 2011, DWP started to reassess everyone who receives Incapacity Benefit using the Employment and Support Allowance.

### Construction of the variable used in the analysis

SAMHI has been constructed at the Lower Super Output Area level, the smallest area level available in England. On average, there are 4.8 LSOAs in one MSOA. We aggregate the index to the geographical level of our outcome, MSOA level. The aggregation to the MSOA level is done averaging the SAMHI values of the LSOAs within each MSOA. In order to account for the different sizes of LSOAs and MSOAs, we use as weights the population within each LSOA. Our measure is a mean weighted by the population.

# Additional results

# Balanced panel results

Table D.1: Association between log GDHI per capita and SAMHI for a balanced panel

|  | | | Coeff (SE) | | 95% CI | Obsv. (N*T) | MSOAs (N) |
| --- | --- | --- | --- | --- | --- | --- | --- |
| Main variable of interest: SAMHI (sd) | | |  | | | | |
| (1) Baseline Controls (B) + Year FE | | | 0.163 | (0.008) | [0.147;0.180] | 61,101 | 6,789 |
| (2) B + Year FE + MSOA FE | | | 0.022 | (0.003) | [0.017;0.028] | 61,101 | 6,789 |
| Main variable of interest: Lag SAMHI (sd) | | |  | | | | |
| (3) B | + | Year FE + MSOA FE | 0.019 | (0.003) | [0.014;0.025] | 54,312 | 6,789 |
| (4) B | + | MSOA FE + YEAR x Region FE | 0.012 | (0.002) | [0.008;0.016] | 54,312 | 6,789 |
| (5) B | + | MSOA FE + YEAR x TTWA FE | 0.009 | (0.002) | [0.005;0.013] | 54,306 | 6,789 |
| (6) B | + | MSOA FE + YEAR x LAD FE | 0.006 | (0.002) | [0.002;0.010] | 54,312 | 6,789 |

# Notes: Each column displays the coefficients for the component of SAMHI variable in the regression. The specification of the regressions is indicated at the beginning of end of the column line. Clustered standard errors in parentheses at the LAD level. Controls include population age groups, LAD unemployment rate and LAD percentage of population with no qualification. FE: fixed effects; MSOA: Middle Super Output Area; TTWA: Travel To Work Area; LAD: Local Authority District. N*T is the number of observations in the regressions, MSOAs (N) the number of MSOAs contributing to the regression

# Different temporal lags results

Table D.2: Association between log GDHI per capita and different temporal lags of SAMHI

|  | (1) (2)  Log deflated GDHI per capita | |
| --- | --- | --- |
| Lag SAMHI | 0.011 | 0.013 |
| (se) | (0.003) | (0.003) |
| 95% CI | [0.006;0.016] | [0.007;0.019] |
| Lag 2 SAMHI | 0.009 | 0.005 |
| (se) | (0.003) | (0.002) |
| 95% CI | [0.003;0.014] | [0.001;0.010] |
| Lag 3 SAMHI |  | 0.005 |
| (se) |  | (0.003) |
| 95% CI |  | [0.000;0.010] |
| controls | YES | YES |
| year FE | YES | YES |
| MSOA FE | YES | YES |
| Obsv. (N*T) | 43,830 | 37,160 |
| MSOAs (N) | 6,782 | 6,738 |

Notes: Each column displays the coefficients for the component of SAMHI variable in the regression. The specification of the regressions is indicated at the beginning of end of the column line. Clustered standard errors in parentheses at the LAD level. Controls include population age groups, LAD unemployment rate and LAD percentage of population with no qualification. FE: fixed effects; MSOA: Middle Super Output Area; TTWA: Travel To Work Area; LAD: Local Authority District. N*T is the number of observations in the regressions, MSOAs (N) the number of MSOAs contributing to the regression.

# Different SAHMI components results

Table D.3: Association between log GDHI per capita and different components of SAMHI

|  | (1) (2) (3) (4)  Log deflated GDHI per capita | | | |
| --- | --- | --- | --- | --- |
| Lag antidep | -0.032 |  |  |  |
| (se) | (0.004) |  |  |  |
| 95% CI | [-0.039;-0.025] |  |  |  |
| Lag depression |  | -0.005 |  |  |
| (se) |  | (0.001) |  |  |
| 95% CI |  | [-0.008;-0.002] |  |  |
| Lag IBESA |  |  | -0.012 |  |
| (se) |  |  | (0.003) |  |
| 95% CI |  |  | [-0.017;-0.006] |  |
| Lag hospital admission |  |  |  | -0.006 |
| (se) |  |  |  | (0.002) |
| 95% CI |  |  |  | [-0.010;-0.002] |
| controls | YES | YES | YES | YES |
| year FE | YES | YES | YES | YES |
| MSOA FE | YES | YES | YES | YES |
| Obsv. (N*T) | 50,448 | 50,448 | 50,448 | 50,448 |
| MSOAs (N) | 6,789 | 6,789 | 6,789 | 6,789 |

Notes: Each column displays the coefficients for the component of SAMHI variable in the regression. The specification of the regressions is indicated at the beginning of end of the column line. Clustered standard errors in parentheses at the LAD level. Controls include population age groups, LAD unemployment rate and LAD percentage of population with no qualification. FE: fixed effects; MSOA: Middle Super Output Area; TTWA: Travel To Work Area; LAD: Local Authority District. N*T is the number of observations in the regressions, MSOAs (N) the number of MSOAs contributing to the regression.


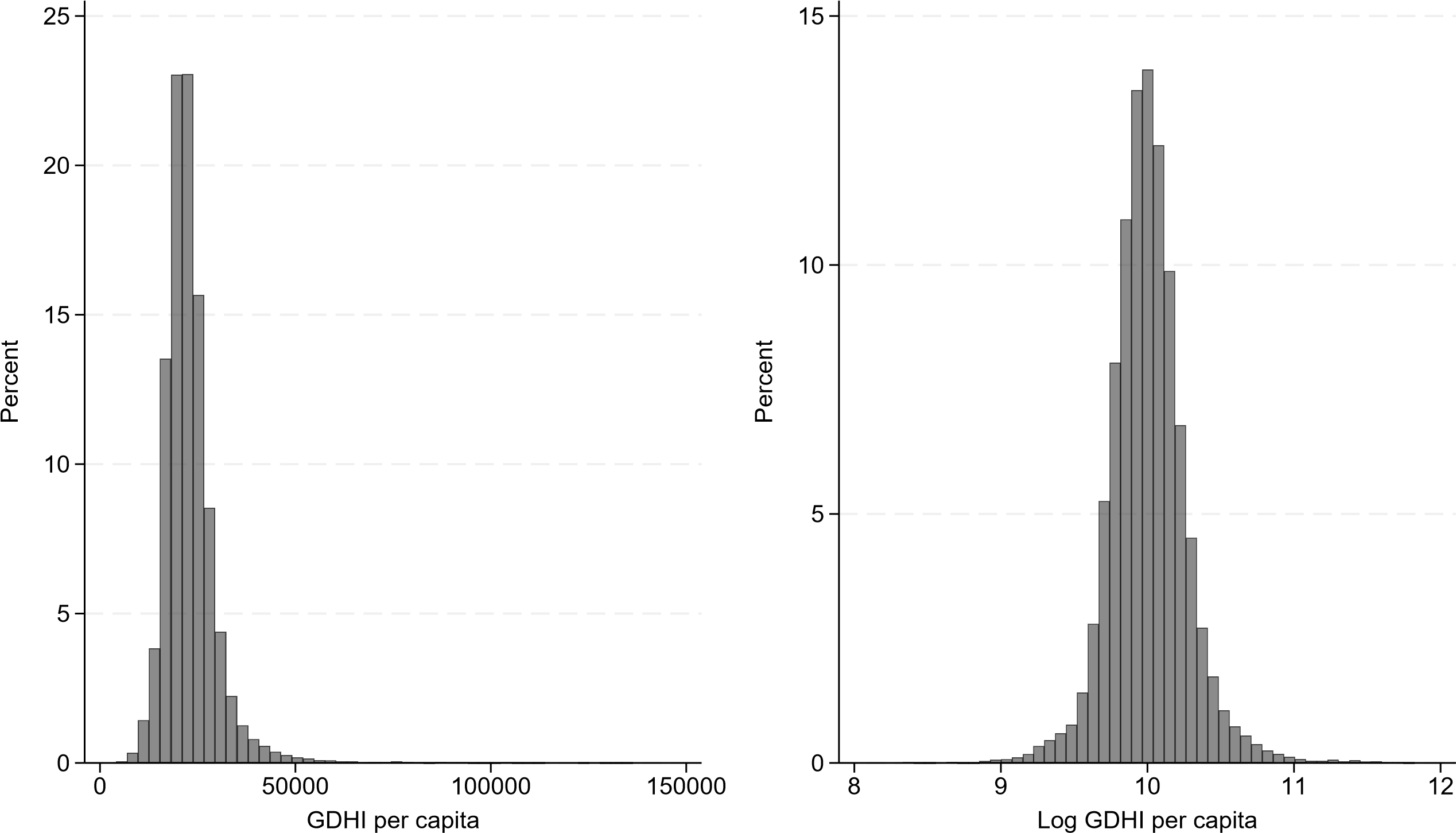


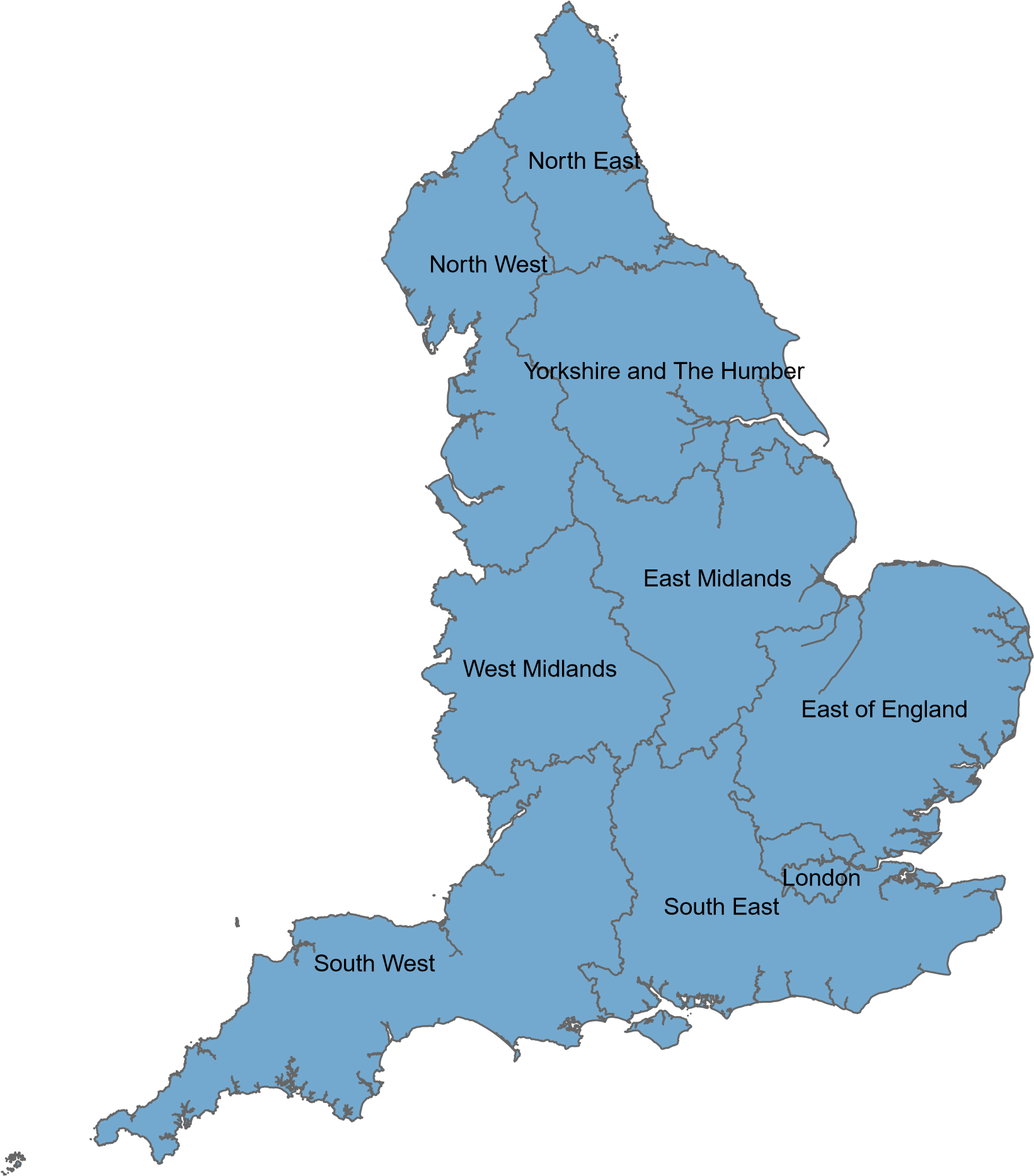

Supplement: online supplemental file 1 [file bmjopen-16-6-s001.docx]
