# Supplementary material for: Is there an association between mental health and economic prosperity? A longitudinal ecological study in England, 2011–2019
Source: BMJ Open. 2026 Jun 8;16(6):e113549. doi: 10.1136/bmjopen-2025-113549 (PMC13250192; doi:10.1136/bmjopen-2025-113549)
Supplement: Supplementary file 2 [file bmjopen-2025-113549.reviewer_comments.pdf]

PEER REVIEW HISTORY

BMJ Open publishes all reviews undertaken for accepted manuscripts. Reviewers are asked to complete a checklist review form and are provided with free text boxes to elaborate on their assessment. These free text comments are reproduced below.

ARTICLE DETAILS

Title (Provisional)

Is there an association between mental health and economic prosperity? A longitudinal ecological study in England, 2011-2019

Authors

Mignon, Doriane; Khavandi, Sam; Bambra, Clare; Sutton, Matt; Munford, Luke A.

VERSION 1 - REVIEW

Reviewer 1

Name Beazley, Peter

Affiliation University of East Anglia, Clinical Psychology and Psychological Therapies

Date 02-Dec-2025

COI For full disclosure I have identified one paper in the peer review report which is my own publication but I feel this is germane to the authors. I do not think they need to cite it necessarily but it seems very relevant to critiquing part of their argument.

I have no other competing interests to declare.

General

Thank you for the opportunity to review this interesting paper. It addresses an important question, and I have plenty of feedback for the authors to consider. However fundamentally I have some wider issues which may potentially locate my recommendation of 'major revision' as a generous suggestion:

• If the paper can only test an association and not identify directions of causality, to what degree is the work genuinely novel, or to what degree does it make a large contribution in terms of policy? General relationships between economic activity and mental health are well established. What seems less well understood are mechanisms on the individual level.

* If the work is aiming to test directions of causality, and clearly to some degreee it is, then the authors need to in my view be much clearer about this and why they are convinced the directions of causality do not run the other way.

• The authors haven't, in my view, engaged with some of the fairly clear limitations of the SAMHI and what this actually represents, and also perhaps how what it represents might have changed over time. See comments below. I acknowledge they note this is the limitations section but this feels very fundamental. I am genuinely not persuaded by how meaningful the SAMHI is as a measure especially one used to look at longitudinal change.

• Much of the report is rather technical; the analysis is unlikely to be familiar or readily comprehensible to a clinician, and this creates a risk that the headlines of the paper are used to justify things that the data do not necessarily show – see comments above and below.

Introduction

• Need to explain Tekin reference, Page 2 line 34 and why you indicate results are imprecise even if briefly – this can't just appear as an unjustified statement.

• I was surprised that the argument only really focused on the direction of relationship between mental health and economic performance (or the fact there is a relationship without addressing its directionality). There must surely be bi-directional effects. Explaining briefly why effects might occur in both directions would help contextualise the work.

• I would like the authors to consider how to strengthen the argument of why it is interesting, necessary or important to review the relationship at a small 'sub national geographical level'. The only argument appears to be that this hasn't been looked at. But why do the authors think this is interesting or important to do so? This is also important in terms of the editorial decision of relevance etc.

Method/Results

• This comes in the limitations section towards the end, but I think the point about what the SAMHI actually represents could be more critically engaged with in the study. For a start, I would suggest it is important to be clear throughout the manuscript that the SAHMI is not really a measure of 'mental health' but 'mental health service utilisation and benefit payments' at a population level (I'm not suggesting to use this specific term in quotes, but you see what I mean). Service utilisation of course depends on availability. But there are large scale population differences in mental health service availability (for example see Beazley 2024 https://doi.org/10.12968/bjmh.2024.0018 which actually highlights that during the period of the interest of the present study i.e. 2011-2019 there was a net reduction in mental health workforce in line with the period of austerity. However, the nature of the change in the workforce is geographically uneven. The 'Paying the Price' report by the King's Fund in 2008 is also relevant here as a forecast for the period of study, as is this report in this journal by Wang et al https://doi.org/10.1136/bmjopen-2023-073731). There are also large differences in prescribing practices for antidepressants (for example Heald et al 2020 https://doi.org/10.1002/hup.2741) , and no doubt factors such as urbanicity or rurality make a difference to likelihood of hospital admissions and management of pre-admission

mental health crises. Moreover, in some respects higher service utilisation may actually reflect better individual level mental health as it may suggest fewer people are not receiving treatment or appropriate intervention (at least in relation to depression prevalence/anti dep use).

• In a similar vein, the elements of the SAMHI are likely to have changed differently over time. Antidepressant use has no doubt shot up (presumably meaning lower SAMHI scores in your analysis). Depression may have increased more modestly (ditto). ESA payments – not sure, but there was a clear policy intention to reduce these (and if so, would that have led to increased scores on the SAMHI). Hospital admissions have also probably reduced depending on how these are defined (ditto). Overall, therefore, the composition of the SAMHI is likely to be rather different in 2019 than in 2011, with the different factors moving in different directions. If mental health defined by the SAMHI has got worse over time, I wonder if that is because of the presumed outsized contribution of antidepressant prescribing increases?

• I've written a lot on this point but it seems to me that engaging critically with the meaning of the SAMHI and what it represents seems, to me, to be a key step for a sensible interpretation of the results, and fairly fundamental to the paper – needing more consideration than just a limitation comment. Maybe, the validity and meaningfulness of the SAMHI is just not enough to make this a particularly meaningful analysis.

• Table 2 – I can see the models are referred to as 'lines' which is confusing. I was expecting a graph! I can see you just mean the lines on the table. Unless there is a technical meaning I'm unaware of please

Discussion

• I am really not convinced how strong a control the use of lagged values will have been for teasing out directions of causality. The relationships between these variables are complex, and assumes that the mental health at time points before t-1 is not relevant. This feels rather ropey. Moreover, surely SAMHI scores are overall temporally relatively stable. In essence this approach feels like it makes quite a lot of assumptions even in terms of a population based analysis, and I would suggest the authors need to account for this more transparently in their discussion and indeed wider analysis. If I have missed something in the interpretation then perhaps this is a case of clarifying it, but if these criticisms are reasonable then the questions of directionality need better consideration.

• I would invite the authors to engage with the magnitude of their results a bit more and be careful in other interpretations. A standard deviation increase in SAMHI is likely to be a very noticeable change whether on a population or individual level; but a 1.9% increase in GDHI whilst of course meaningful at lower ends of income spectrum is much smaller (what, about 6 months of inflation currently?). The proposed suggestion of a one standard deviation increase in SAMHI for a population basis is surely an enormous change, and it begs the

question – what level of intervention (and therefore cost/investment) would be required to achieve such an increase? Presumably we could achieve it also through unhelpful means e.g. axing welfare payments for MH or anything which led to SSRI prescriptions being restricted. On one hand, could a critical policymaker interpret your findings entirely differently – e.g. that there's no point investing into improving mental health, as the economic benefits are overblown? Ultimately, I'm somewhat concerned that the findings might promote slightly misguided and simplistic policy assumptions which don't exist in the data.

• Moreover, if you're making an argument here you about this you need to be careful about your population reference: 56.3 million will include children and older adults who are economically inactive or who have no means of increasing their GDHI which they control.

Small Elements

Typo Page 1 – Line 42 – "over time" not "overtime"; also page 4, line 45 and line 50. Please check whole manuscript.

**Reviewer**	**2**
**Name**	**McManus, Sally**
**Affiliation**	**City University of London**
**Date**	**13-Dec-2025**
**COI**	**None**

This is a neat, focused contribution to the field. While we're used to looking at economic circumstances contributing to subsequent mental health outcomes, this more makes a case using area-level data for an association between aggregated population mental health and subsequent (area-level) economic outcomes. Potential bidirectionality of the association is clearly acknowledged in the limitations.

Language varies with discipline and sector, the word 'longitudinal' in some settings tends to have a specific meaning denoting repeated measures on the same individuals rather than on repeated cross-sections of the same areas (with their changing populations). This area-level analysis is also perfectly valid, but personally I was confused by and would suggest dropping the word 'longitudinal' from the title.

Positive mental health is constructed here as the absence of mental illness– given the amount of work this century making the case that positive mental health is not simply the absence of mental illness, was there a reason for not drawing on the measures of positive mental wellbeing LAs collect? What is done is fine, just worth noting the distinction between lack of mental illness and positive mental health.

It might be worth mentioning the significant opposite direction of association for London in the discussion? Agree it should not be singled out as a focus, but it is interesting?

You note how: “There is a negative trend indicating more healthcare use for mental health reasons overtime, which we interpreted as a worsening of population mental health. One potential competing explanation could be that as norms have been evolving and the population has become more aware of mental health issues, healthcare use for mental health issues has been increasing. Given that the index relies on serious mental health issue measures, one could assume that this is not the case. Serious mental health issues require professional treatment, and one would not expect the approach of these by health professionals to have been varying overtime”

I suggest rewording this, this interpretation is likely wrong – please see this chapter which shows the proportion of people depression or an anxiety disorder receiving treatment (medication or talking therapy) has increased steeply over time:

Clery, E., Morris, S., Wilson, C., Cooper, C., Das-Munshi, J., McManus, S., & Weich, S. (2025). Mental health treatment and service use. In Morris, S., Hill, S., Brugha, T., McManus, S. (Eds.), (2025) Adult Psychiatric Morbidity Survey: Survey of Mental Health and Wellbeing, England, 2023/4. NHS England. https://digital.nhs.uk/data-and-information/publications/statistical/adult-psychiatric-morbidity-survey/survey-of-mental-health-and-wellbeing-england-2023-24/mental-health-treatment-and-service-use

The appendices are thoughtful and detailed, its written clearly and well, and I think it’s clear focus on a reframing of the direction of association makes this a valid and interesting contribution.

Minor

Page 3: “associations with other health outcomes e.g. smoking, drinking, physical activity…” would describe smoking, drinking and activity as health behaviours rather than outcomes.

**Reviewer**	**3**
**Name**	**Dack, Kyle**
**Affiliation**	**University of Bristol, MRC Integrative Epidemiology Unit**
**Date**	**19-Jan-2026**
**COI**	**None**

Statistical review summary:

Overall, the core analysis is sound, and the interpretation is aligned with the data and assumptions. However, currently methods/results reporting assumes an econometric background which readers may not possess. There are also important details missing in terms of measurement methods, exclusion criteria, and modelling strategy. I recommend applying STROBE, which may help to address these issues.

Abstract:

1. The abstract is clear but it lacks some key details which may help prepare readers for the full text, in particular – how is “mental health” quantified, the model is applied to estimate the association, and the total “N” (i.e. number of MSOA).

Introduction:

2. Is a bidirectional relationship being proposed? The introduction notes that mental health can deteriorate due to economic factors, but also that mental health can be a causal factor of future income (via labour market exclusion). There is evidence to support this, but it may confuse readers because it is not explicitly stated that the effect could be two-way (i.e. present mental health affects future economic status, present economic status affects future mental health).

-I also suggest clarifying the temporal sequence in the stated aim – the association between mental health and future economic prosperity (at a population level).

Methods:

3. A reporting checklist was not included with the manuscript – I suggest that STROBE would be mostly suitable, because this is an ecological observational study.

4. The Data subsection gives a good overview of the key characteristics of the dataset, but not where it was sourced from. This is provided in later sections, but it may make more sense under “Data”.

5. Exclusion criteria: Is it possible to give more information about the criteria used to exclude the two MSOA due to population and outcome values?

6. Outcome: There are a few elements which make it difficult to fully interpret this section and I Suggest considering:

-While details of how the outcome was measured in the original data are provided in C1, a brief summary in the main text would be convenient for readers.

-“we used the log to interpret our results as changes in percentage” – suggest rephrasing as “we log-transformed prior to modelling so that coefficients could be interpreted as percentage changes”, or similar, to be more precise.

-How the outcome is handled temporally. i.e. that it was observed annually 2011-2019, with the intention of modelling with the prior year of mental health. What was the rationale for a 1 year time lag rather than say, 2 or more years?

7. SAMHI: Is it possible to report the range of the original score, and what 1 SD unit corresponds to? This would aid with interpretation.

8. Model: The modelling approach is reasonable and I have no concerns regarding its application. However, I do suggest adding a few details which may help readers follow, particularly those without an econometrics background:

-Explicitly state that this is a repeated measures analysis, i.e. repeated observations of the same MSOA at different timepoints.

-The rationale for fixed effects at the area level is clear and accurate, but it may also help to state that this has the effect of accounting for non-independence from repeated measures.

-Using the 1 year time lagged exposure has a strong rationale – to create a temporal separation which allows for a stronger interpretation of the association (compared to cross-sectional analysis). However, it would be more precise to avoid stating it accounts for reverse causality, because past GDHI can still affect future mental health and is not estimated in this analysis. Instead, it reduces reverse causality as a concern when interpreting the association of interest.

Results:

9. For each table I suggest adding the N of MSOA contributing to the statistics.

10. “The mean of the SAMHI over the period is 0” isn’t it 0.02? The range of possible values seems to be 0-1, so I recommend not rounding it. In the sentences discussing this it may help readers to remind them that higher values reflect better area-level mental health.

11. Figure 1a / 2b. I suggest reporting the definitions of 1-4, e.g. are these quartiles?

12. Table 2: There are elements of the analysis indicated in this table which were not reported in the methods and impair readability or otherwise seem unclear:

13. -The methods should report that there are multiple models, as currently it gives the impression of a single model, and include the rationale for each. Particularly the unlagged models, these are cross-sectional and have poorer interpretability than the longitudinal models, and it is not clear how this aligns with the aims?

-The N varies per model, is this due to missing data? If so I suggest adding a line to report this in the statistical analysis.

-Please add the units of both the exposure and outcome to ensure the coefficient is interpreted accurately.

-There is reference to “baseline controls” but this was unmentioned in the methods.

-The statistics are reported on a 0-1 scale in the table, but then in a 0-100 (i.e., percentages scale) in the text, which may confuse readers.

Discussion

14. “The longitudinal nature of our data enabled us to control for unobservable characteristics at the area level.” I think it is important to qualify this with a note that it was the MOSA fixed effects which enabled control of unobserved characteristics, and this has the assumption that the confounders are constant over time (time-invariant).

15. Limitations: I suggest that “reverse causality” may not be the precise issue. Typically, reverse causality would refer to a true effect of GDHI -> SAMHI contaminating the SAMHI -> GDHI association, if both were measured at the same timepoint.

-In this study, the association estimated is SAMHI_year_-1 -> GDHI_year_0, which would rule out reverse causality.

-The authors raise the concern that perhaps past GDHI could influence mental health. E.g. “GDHI_year_-2 -> SAMHI_year_-1 -> GDHI_year_0”, as well as past GDHI affecting future GDHI: “GDHI_year_-2 -> GDHI_year_-1 -> GDHI_year_0”

-However, this is more reflective of bidirectional causality and dynamic confounding. In practical terms it could mean that the association of interest is in part reflective of unmodelled income dynamics. I suggest the authors consider this and if they agree, to rephrase the present limitation, because the phrasing is a little imprecise.

VERSION 1 - AUTHOR RESPONSE

RESPONSE TO REVIEWER 1

We thank you for the thoughtful and detailed review. We have carefully revised the manuscript to address each point. Below, we provide a point-by-point response with proposed text changes (in quotation marks and bold) and a summary of analyses and robustness checks we have added. Page/section references correspond to the revised manuscript.
We hope these revisions address the reviewer’s concerns and improve the clarity, transparency and policy relevance of the manuscript.

Summary of major changes
• Clarified our study’s aim (association, not causation) and rephrased policy claims throughout, including the Abstract and Discussion. Added explicit caveats and avoided causal language.
• Expanded the Introduction to explain bidirectionality between mental health and economic conditions, and clarified the Tekin et al. (2013) finding and why we characterised their estimates as imprecise.
• Substantially strengthened our description and critical appraisal of the Small Area Mental Health Index (SAMHI): what it captures (service utilisation/benefit receipt/diagnosed prevalence) versus what it does not (true prevalence), potential sensitivity to supply and policy, and possible composition changes over time. Added citations and expanded Limitations subsection moved earlier in the Methods/Measures section. We have adapted the text in the paper to refer more consistently to ‘service utilisation related to mental ill-health' and ‘proxy of population mental health'.
• Added new sensitivity analyses (which are reported in the Appendix): (i) balanced panel results (ii) introducing extra lag of the small area mental health index (iii) different regressions with each component of the small mental health area index
• Renamed “lines” in Table 2 to “models” and clarified the specification in the footnote. Corrected typographical errors (“over time”, etc.) and harmonised terminology.
• Reframed magnitude interpretation: reported effects for smaller, policy-relevant shifts (e.g., 0.1 or 0.2 SD) and avoided “national total” extrapolations that could be misinterpreted. Added context on who is included in GDHI per capita and implications for interpretation.

General comments

Comment: If the paper can only test an association and not identify directions of causality, to what degree is the work genuinely novel, or to what degree does it make a large contribution in terms of policy? General relationships between economic activity and mental health are well established. What seems less well understood are mechanisms on the individual level.

* If the work is aiming to test directions of causality, and clearly to some degree it is, then the authors need to in my view be much clearer about this and why they are convinced the directions of causality do not run the other way.

Response: We fully agree that our design does not permit strong causal inference, and we have clarified this more explicitly throughout the manuscript. Our intention is not to claim causality but to provide new evidence on within-area longitudinal associations at a much finer spatial scale than previously examined. While relationships between health and economic activity are well documented at national and individual levels, evidence at subregional geographies—particularly using a consistent annual index of area level mental health need—is limited. Our contribution is therefore empirical rather than causal: we exploit nine years of panel data to examine whether changes in area level mental health are followed by changes in area level disposable income, controlling for rich sets of fixed effects and time varying covariates.

To avoid any misunderstanding, we have revised the Introduction, Methods, and Discussion to:

1. State explicitly that our analysis is associational, not causal.
2. Explain why lagging the SAMHI is used only as a temporal ordering test, not as an identification strategy.
3. Acknowledge that reverse causality is plausible and may still bias estimates despite lagging.
4. Clarify the nature of the novelty—the use of a unique small area mental health index, linked longitudinally to economic prosperity at MSOA level, enabling examination of spatial heterogeneity across English regions.

We believe these revisions strengthen the paper by aligning our claims more clearly with what the data can support.

Manuscript changes related to this point:

- Introduction, at the end of page 2 beginning of page 3, new paragraph:

"**In this study, however, we focus on one temporal sequence only—the association between changes in population mental health and subsequent changes in area-level economic prosperity. The analysis is therefore directional only in terms of timing and should not be interpreted as establishing causal effects. Our objective is to describe these temporal associations at a fine spatial scale, rather than to estimate causal impacts. While the direction of causality between mental health and economic outcomes has been examined extensively at national and individual levels, there is limited evidence on how such dynamics operate across small geographic areas. Studying the association between health and economic prosperity at a small area level has several strengths for this study. The granularity of the data allows to understand the influence of fine-grained environmental and socioeconomic factors. Particularly relevant to our case, it allows to detect local health inequalities that larger geographies obscure and provide emerging evidence for designing and targeting interventions with much greater precision. By using annual MSOA-level data over nine years, we contribute new empirical evidence on temporal associations across local areas in England, while recognising that the data do not support causal inference.** "

- Methods, section statistical analysis, page 4 to 5:

"**The first model (model 1) included year fixed effects and baseline controls. It provided us with a baseline of the association between SAMHI and the log of GDHI per capita. This model is likely biased as it does not account for unobservable time invariant characteristics at the area level which are likely to confound the association. In model 2, we added fixed effects at the area**

(MSOA) level to account for unobservable time invariant characteristics. The association is still likely to be biased as it might capture reverse causality, i.e. that economic prosperity causes population mental health. As we wanted to test the other direction, i.e. that population mental health causes economic prosperity, we use the variable SAMHI of the previous time period to introduce a temporal ordering and reduce the concern for reverse causality. It is important to note that including lagged SAMHI values does not identify a causal effect or remove reverse causality. Economic conditions may influence mental health over multiple past periods, and SAMHI itself is relatively persistent over time. The lag specification therefore serves only to examine temporal ordering, not to isolate exogenous variation. As such, the models estimated associations within areas, and the directionality of the relationship cannot be definitively established. "

- Discussion, paragraph Strengths and weaknesses of the study, towards end of page 9:

"**However, we could not entirely rule out the possibility that the significant positive coefficient is due to bidirectional causality and dynamic confounding, i.e. that past GDHI per capita affect both SAHMI in the subsequent period and GDHI per capita in the subsequent period. Modelling this dynamic is out of the scope of this study, but it means that our estimates may be biased and probably an upper bound of the true effect of past mental health use on subsequent GDHI per capita.**"

Introduction related comments

Comment: Need to explain Tekin reference, Page 2 line 34 and why you indicate results are imprecise even if briefly – this can't just appear as an unjustified statement.

Response: We have now added that the authors qualify their coefficients as imprecise and that they attribute this in part to measurement problems of the mental health variable. The sentence now reads: "**What has been documented -using both aggregate and micro- level data- is the association between economic growth and mental health, with deteriorations in mental health often linked to recessions [8, 9, 10, 11], although Tekin et al. [12], using microdata, find no significant association (the authors qualify their results as imprecise which they link to potential measurement problems).**" (second paragraph of introduction, page 2)

Comment: I was surprised that the argument only really focused on the direction of relationship between mental health and economic performance (or the fact there is a relationship without addressing its directionality). There must surely be bi-directional effects. Explaining briefly why effects might occur in both directions would help contextualise the work.

Response: We have added a new paragraph outlining plausible bidirectional pathways (economic downturns affecting mental health via income insecurity/unemployment; and mental ill-health reducing employment, hours, and productivity) and why our empirical strategy focuses on temporal ordering and fixed effects rather than establishing causality. This context motivates our use of lagged SAMHI and regional heterogeneity analysis.

Manuscript change:

In last paragraph of introduction on page 2: "**The wider literature highlights a bidirectional relationship between economic conditions and mental health: economic circumstances can shape subsequent mental health outcomes, while mental health itself can influence economic prosperity. Adverse economic conditions can worsen mental health through income loss, unemployment and insecurity, while mental ill-health can reduce labour market participation and productivity, with area-level consequences for household income. In this study, however, we focus on one temporal sequence only—the association between changes in population mental health and subsequent changes in area-level economic prosperity. The analysis is therefore directional only in terms of timing and should not be interpreted as establishing causal effects.**

Our objective is to describe these temporal associations at a fine spatial scale, rather than to estimate causal impacts. While the direction of causality between mental health and economic outcomes has been examined extensively at national and individual levels, there is limited evidence on how such dynamics operate across small geographic areas."

Comment: I would like the authors to consider how to strengthen the argument of why it is interesting, necessary or important to review the relationship at a small 'sub national geographical level'. The only argument appears to be that this hasn't been looked at. But why do the authors think this is interesting or important to do so? This is also important in terms of the editorial decision of relevance etc.

Response: Our contribution is threefold: (1) we assemble and analyse an unusually granular, longitudinal small-area panel (6,789 MSOAs over 2011–2019) to quantify the association between a validated area-level mental health index and GDHI, controlling for MSOA fixed effects and rich time-varying geography-by-year effects; (2) we map and quantify substantial regional heterogeneity in the association; and (3) we transparently bound claims and provide robustness checks, including lagged exposure, decomposition of the index.

We proposed the following manuscript change in the introduction to make these contributions explicit (start end of page 2 and finish beginning of page 3): "**While the direction of causality between mental health and economic outcomes has been examined extensively at national and individual levels, there is limited evidence on how such dynamics operate across small geographic areas. Studying the association between health and economic prosperity at a small area level has several strengths for this study. The granularity of the data allows to understand the influence of fine-grained environmental and socioeconomic factors. Particularly relevant to our case, it allows to detect local health inequalities that larger geographies obscure and provide emerging evidence for designing and targeting interventions with much greater precision. By using annual MSOA-level data over nine years, we contribute new empirical evidence on temporal associations across local areas in England, while recognising that the data do not support causal inference.** "

Method/Results related comments

Comment: This comes in the limitations section towards the end, but I think the point about what the SAMHI actually represents could be more critically engaged with in the study. For a start, I would suggest it is important to be clear throughout the manuscript that the SAHMI is not really a measure of 'mental health' but 'mental health service utilisation and benefit payments' at a population level (I'm not suggesting to use this specific term in quotes, but you see what I mean). Service utilisation of course depends on availability. But there are large scale population differences in mental health service availability (for example see Beazley 2024 https://doi.org/10.12968/bjmh.2024.0018 [doi.org] which actually highlights that during the period of the interest of the present study i.e. 2011-2019 there was a net reduction in mental health workforce in line with the period of austerity. However, the nature of the change in the workforce is geographically uneven. The 'Paying the Price' report by the King's Fund in 2008 is also relevant here as a forecast for the period of study, as is this report in this journal by Wang et al https://doi.org/10.1136/bmjopen-2023-073731 [doi.org]). There are also large differences in prescribing practices for antidepressants (for example Heald et al 2020 https://doi.org/10.1002/hup.2741 [doi.org]) , and no doubt factors such as urbanicity or rurality make a difference to likelihood of hospital admissions and management of pre-admission mental health crises. Moreover, in some respects higher service utilisation may actually reflect better individual level mental health as it may suggest fewer people are not receiving treatment or appropriate intervention (at least in relation to depression prevalence/anti dep use).

Comment: In a similar vein, the elements of the SAMHI are likely to have changed differently over

time. Antidepressant use has no doubt shot up (presumably meaning lower SAMHI scores in your analysis). Depression may have increased more modestly (ditto). ESA payments – not sure, but there was a clear policy intention to reduce these (and if so, would that have led to increased scores on the SAMHI). Hospital admissions have also probably reduced depending on how these are defined (ditto). Overall, therefore, the composition of the SAMHI is likely to be rather different in 2019 than in 2011, with the different factors moving in different directions. If mental health defined by the SAMHI has got worse over time, I wonder if that is because of the presumed outsized contribution of antidepressant prescribing increases?

Comment: I've written a lot on this point but it seems to me that engaging critically with the meaning of the SAMHI and what it represents seems, to me, to be a key step for a sensible interpretation of the results, and fairly fundamental to the paper – needing more consideration than just a limitation comment. Maybe, the validity and meaningfulness of the SAMHI is just not enough to make this a particularly meaningful analysis.

Summary of comments related to the small area mental health index (SAMHI) (from general comments): The authors haven't, in my view, engaged with some of the fairly clear limitations of the SAMHI and what this actually represents, and also perhaps how what it represents might have changed over time. [...] I acknowledge they note this is the limitations section but this feels very fundamental. I am genuinely not persuaded by how meaningful the SAMHI is as a measure especially one used to look at longitudinal change

Response: The Reviewer raises an important concern about what SAMHI represents and how changes in service availability, prescribing practices, and benefit policy might affect its interpretation over time.

We fully agree that SAMHI is not a direct measure of population mental health but rather a composite indicator reflecting multiple manifestations of population-level mental health need, including service utilisation, prescribing, and benefit claims. To avoid overstating the construct, we have revised the manuscript to make this distinction explicit in the Methods and Discussion sections.

To address the reviewer's concerns, we have:

1. Expanded the description of SAMHI to emphasise what it does and does not measure.
2. Added a detailed discussion of time-varying service availability, prescribing trends, and benefit policy reforms, and how these might influence SAMHI.
3. Clarified that our inferences concern changes in a composite proxy for population mental health need, not clinical incidence
4. Added a paragraph in the method section ("A**ccounting for limitations of Small Area Mental Health Index (SAMHI)**" (page 5) describing how our statistical analysis account for these limitations

We have additionally substantially expanded our description of SAMHI. We explicitly describe its components and construction; clarify that it is a composite index drawing on service utilisation (hospital attendances), diagnosed prevalence (QOF depression), prescribing (antidepressants) and benefit receipt (IB/ESA for mental illness); and emphasise that it may be sensitive to service availability, clinical practice, and welfare policy. We move core limitations from the end to the Measures section and report estimates for each component separately as a sensitivity check.

We have also adjusted our terminology to avoid presenting the index as a direct measure of population mental health. Instead, we emphasise that it reflects use of services for mental-ill-health–related reasons, and that the factor-analysis approach identifies an underlying construct which we interpret as population mental health. Consequently, the index serves as a proxy for population mental health rather than a direct measure. We have aimed to make this distinction explicit throughout the manuscript, and any remaining instances where SAMHI is referred to as population mental health are unintentional oversights on our part.

Changes in the manuscript:

- In method – main variable of interest (page 3):

"SAMHI is a composite indicator intended to capture area-level mental health through multiple administrative measures related to service use, prescribing, and disability claims. It therefore reflects both underlying mental health burden and patterns of service utilisation, which are themselves shaped by service availability, clinical practice, and policy context. This index "is proportional to the overall burden on the healthcare system" [14] but is not an indicator of the prevalence of population mental health."

- In method – statistical analysis (page 5):

"Accounting for limitations of Small Area Mental Health Index (SAMHI)

SAMHI is a composite indicator of area-level reflecting the use of services (healthcare, prescription, benefits) for mental-ill-health-related reasons. The construction of the index relies on factor analysis and the weights attributed to each components indicate how they each contribute to an underlying construct, which is interpreted as population mental ill-health in the area. Because the SAMHI relies on use of service which may vary with service supply, clinical practice and social security policy as well as underlying morbidity, the components can evolve differently overtime and by geographical level. We account for this by (a) extensive fixed effects and geography-by-year controls (models 4 to 6), and (b) sensitivity analyses where we present the results for each component of the SAMHI separately (results in Appendix D.3). The fixed effects in models 4 to 6 would control for the possibility that SAMHI is different depending on characteristics which are not varying overtime at the geographical level (such as rural/urban) and that the trend in the SAMHI can be different by regions (e.g. the level of service use different regionally overtime). "

- In discussion – in strengths and weaknesses of the study - first paragraph commencing on page 10:

"SAMHI's strengths are small-area coverage and multi-source design, but three limitations are salient. First, SAMHI is a proxy that integrates service use, prescribing and benefit claiming; each component is sensitive to local availability, clinical practice, and eligibility rules, and thus may diverge from true morbidity. Recent work showed indeed that the proportion of people with depression receiving treatment has increased over time [18]. We therefore avoid framing results as changes in prevalence and instead describe changes in the use of services for mental-ill-health–related reasons. Second, time-varying composition of the index (e.g., strong upward trend in antidepressant prescribing; discontinuation/replacement of benefits series after 2019) complicates the attribution of longitudinal patterns to any single driver [19, 13]. Third, geographical heterogeneity—in workforce, access, and care-seeking attitudes—means that SAMHI may capture unmet need differently across areas [20, 21, 22]. These issues do not invalidate SAMHI's use, but they constrain causal interpretation; we therefore keep policy claims modest and focus on relative area differences and patterning."

Comment: Table 2 – I can see the models are referred to as 'lines' which is confusing. I was expecting a graph! I can see you just mean the lines on the table. Unless there is a technical meaning I'm unaware of please

Response: The text was indeed referring to the lines in the table, but we understand that this might be confusing and thank you for underlining this lack of clarity. We have change to model which is a more appropriate term. We have renamed "lines" to "models" and added a clarifying footnote that each model corresponds to a separate regression with the specification listed in the first column.

Discussion related comments

Comment: I am really not convinced how strong a control the use of lagged values will have been for

teasing out directions of causality. The relationships between these variables are complex and assumes that the mental health at time points before t-1 is not relevant. This feels rather ropey. Moreover, surely SAMHI scores are overall temporally relatively stable. In essence this approach feels like it makes quite a lot of assumptions even in terms of a population-based analysis, and I would suggest the authors need to account for this more transparently in their discussion and indeed wider analysis. If I have missed something in the interpretation then perhaps this is a case of clarifying it, but if these criticisms are reasonable then the questions of directionality need better consideration.
Response: We fully agree that using lagged SAMHI values cannot, on its own, resolve endogeneity or identify causal direction. Our use of the lag was not intended as an identification strategy to establish causality but solely to explore temporal ordering, recognising that both mental health and economic conditions evolve slowly and may influence one another over multiple periods.
To avoid overstating what the model can achieve, we have strengthened the Discussion and clarified in the Method that:

1. Lagged SAMHI does not eliminate reverse causality, because earlier economic conditions may influence mental health at multiple prior time points, not only t–1.
2. SAMHI exhibits relative persistence, meaning the lagged specification is best interpreted as a descriptive temporal association, not a causal estimate.
3. Complex multi-period dynamics between mental health and economic prosperity make it impossible for our design to rule out simultaneous or bidirectional relationships.
4. Our contribution is therefore strictly associational, and we explicitly refrain from causal claims.

We have added text emphasising these points so that the assumptions and limitations of the lagging approach are fully transparent.
We agree that a single lag does not establish directionality, and that SAMHI and GDHI are both persistent. We added some numbers regarding the persistence of the SAMHI within MSOAs in the results section.
Manuscript changes:

- In the method – statistical analysis (page 4 and beginning of page 5):

"**As we are interested in the other direction, i.e. that population mental health causes economic prosperity, we use the variable SAMHI of the previous time period to introduce a temporal ordering and reduce the concern for reverse causality. It is important to note that including lagged SAMHI values does not identify a causal effect or remove reverse causality. Economic conditions may influence mental health over multiple past periods, and SAMHI itself is relatively persistent over time. The lag specification therefore serves only to examine temporal ordering, not to isolate exogenous variation. As such, the models estimated associations within areas, and the directionality of the relationship cannot be definitively established.**"

- In the results – mapping regional inequalities (page 6):

"**The range of the variable is from –6.01 to 2.07 (not shown in table). The standard deviation of 0.92 is mainly differences between MSOAs (between standard deviation is 0.78 - not shown in table), the variation within a MSOA exhibit relative persistence of the index (within standard deviation is 0.47 - not shown in table).**"

- In the discussion – paragraph strengths and weaknesses of the study (page 9):

"**However, we could not entirely rule out the possibility that the significant positive coefficient is due to bidirectional causality and dynamic confounding, i.e. that past GDHI per capita affect both SAHMI in the subsequent period and GDHI per capita in the subsequent period. Modelling this dynamic is out of the scope of this study, but it means that our estimates may be biased and probably an upper bound of the true effect of past mental health use on subsequent GDHI per capita.**"

Comment: I would invite the authors to engage with the magnitude of their results a bit more and be careful in other interpretations. A standard deviation increase in SAMHI is likely to be a very noticeable change whether on a population or individual level; but a 1.9% increase in GDHI whilst of course meaningful at lower ends of income spectrum is much smaller (what, about 6 months of inflation currently?). The proposed suggestion of a one standard deviation increase in SAMHI for a population basis is surely an enormous change, and it begs the question – what level of intervention (and therefore cost/investment) would be required to achieve such an increase? Presumably we could achieve it also through unhelpful means e.g. axing welfare payments for MH or anything which led to SSRI prescriptions being restricted. On one hand, could a critical policymaker interpret your findings entirely differently – e.g. that there's no point investing into improving mental health, as the economic benefits are overblown? Ultimately, I'm somewhat concerned that the findings might promote slightly misguided and simplistic policy assumptions which don't exist in the data. Moreover, if you're making an argument here you about this you need to be careful about your population reference: 56.3 million will include children and older adults who are economically inactive or who have no means of increasing their GDHI which they control.
Response: We now emphasise smaller, empirically (the average within standard deviation of SAMHI is 0.46, i.e. in our sample on average the index varies by 0.46) and policy-relevant changes (e.g., 0.1–0.2 SD) and provide concrete translations at the median GDHI level. We remove the national total extrapolation; instead, we report per-capita and MSOA-level effects and discuss feasible levers. We clarify that GDHI per capita includes all residents (including children and inactive adults), which affects interpretation. We also note that policy changes affecting service utilisation (e.g., prescribing restrictions or disability benefit eligibility) could shift SAMHI without improving underlying mental health; to address this concern, we present component-specific results in appendix D.3.

Manuscript changes:
In the discussion, beginning of second paragraph (page 9): To avoid overstating implications, we report effects for smaller shifts in SAMHI: "**a 0.1 SD increase is associated with ~0.19% higher GDHI per capita (about £44 at the sample mean), and a 0.2 SD increase with ~0.38% (about £87).** "
"**GDHI per capita including all residents (including children and inactive adults), this number is an average which applies to every resident of the region, not only the working population.**"

Small Elements
Comment: Typo Page 1 – Line 42 – "over time" not "overtime"; also page 4, line 45 and line 50. Please check whole manuscript.
Response*:* Thank you for spotting this, the change has been made throughout the document, in multiple locations we replaced "overtime" with "over time".

RESPONSE TO REVIEWER 2
We thank you for the thoughtful and detailed review. We have carefully revised the manuscript to address each point. Below, we provide a point-by-point response with proposed text changes (in quotation marks and bold). Page/section references correspond to the revised manuscript.

Comment: Language varies with discipline and sector, the word 'longitudinal' in some settings tends to have a specific meaning denoting repeated measures on the same individuals rather than on repeated cross-sections of the same areas (with their changing populations). This area-level analysis

is also perfectly valid, but personally I was confused by and would suggest dropping the word 'longitudinal' from the title.
Response*:* We understand that the word longitudinal has been largely use for studies that are following individuals over time. Our understanding of the word longitudinal is that the measures are repeated over time for the same unit. In our case where the unit is an area, we view our study as longitudinal. We would like, for this reason, to keep the title as it is, as we feel the combination 'longitudinal ecological study' is the most precise one***,*** with longitudinal referring to the temporal dimension of our study and ecological referring to the fact that our analysis is based on area level observations.

Comment: Positive mental health is constructed here as the absence of mental illness– given the amount of work this century making the case that positive mental health is not simply the absence of mental illness, was there a reason for not drawing on the measures of positive mental wellbeing LAs collect? What is done is fine, just worth noting the distinction between lack of mental illness and positive mental health.
Response: Thank you for bringing this important topic regarding the distinction to be made between absence of mental illness and positive mental wellbeing. We are aware of the measures of mental wellbeing collected at the Local Authority level; however we wanted to be able to explore mental health at a more granular level and be able to capture the differences within LAs, which can be quite large. To the best of our knowledge, these measures are not available overtime and at our level of interest, Middle Super Output Area. This is a limitation of our work due to data availability. We have made it clearer that our contribution lies in having data at a more granular level. We have also mentioned this limitation in the discussion.
In the discussion (end of Section 'strengths and weaknesses of the study' page 10) we have added the following: "**While a strength of the index is to rely on administrative exhaustive data which relates to services use for mental ill health, we need to be clear that this is capturing only an aspect of population mental health, as the absence of mental ill health does not equal positive mental health. Due to the lack of data of positive mental wellbeing at the geographical level of interest (MSOA), we are limited in that regard, and this remains out of the scope of this study."**

Comment: It might be worth mentioning the significant opposite direction of association for London in the discussion? Agree it should not be singled out as a focus, but it is interesting?
Response: T We have added the following in the discussion to link this explicitly to different regional structural characteristics, potential decreasing returns to scale. We have now in the discussion (page 9): "**Given the existence of substantial regional inequalities, we explored whether the association between mental health and productivity varies across regions. The underlying question was whether investments in mental health would yield similar economic returns across all regions. We do find significantly different coefficients (Figure 3) with most of them (five) being positive and significant, three being non-significant and even the coefficient for London being negative and significant. Regional heterogeneity may reflect differences in structural characteristics or decreasing returns to scale, given that each region starts from a different baseline level of mental health**."

Comment: You note how: "There is a negative trend indicating more healthcare use for mental health reasons overtime, which we interpreted as a worsening of population mental health. One potential competing explanation could be that as norms have been evolving and the population has become more aware of mental health issues, healthcare use for mental health issues has been increasing. Given that the index relies on serious mental health issue measures, one could assume

that this is not the case. Serious mental health issues require professional treatment, and one would not expect the approach of these by health professionals to have been varying overtime"
I suggest rewording this, this interpretation is likely wrong – please see this chapter which shows the proportion of people depression or an anxiety disorder receiving treatment (medication or talking therapy) has increased steeply over time:
Clery, E., Morris, S., Wilson, C., Cooper, C., Das-Munshi, J., McManus, S., & Weich, S. (2025). Mental health treatment and service use. In Morris, S., Hill, S., Brugha, T., McManus, S. (Eds.), (2025) Adult Psychiatric Morbidity Survey: Survey of Mental Health and Wellbeing, England, 2023/4. NHS England. https://digital.nhs.uk/data-and-information/publications/statistical/adult-psychiatric-morbidity-survey/survey-of-mental-health-and-wellbeing-england-2023-24/mental-health-treatment-and-service-use [digital.nhs.uk]
Response: Thank you for bringing to our attention this study. In the light of the results of this and also comments from another reviewer we addressed this in two ways: 1) we have reformulated this paragraph in the discussion and proposed the change of text below and 2) we show in the appendix results for the different components separately to understand whether it appears one component is driving most of the results.

Text change in discussion – in strengths and weaknesses of the study - first paragraph commencing on page 10:
"SAMHI's strengths are small-area coverage and multi-source design, but three limitations are salient. First, SAMHI is a proxy that integrates service use, prescribing and benefit claiming; each component is sensitive to local availability, clinical practice, and eligibility rules, and thus may diverge from true morbidity. Recent work showed indeed that the proportion of people with depression receiving treatment has increased over time [18]. We therefore avoid framing results as changes in prevalence and instead describe changes in the use of services for mental-ill-health–related reasons. Second, time-varying composition of the index (e.g., strong upward trend in antidepressant prescribing; discontinuation/replacement of benefits series after 2019) complicates the attribution of longitudinal patterns to any single driver [19, 13]. Third, geographical heterogeneity—in workforce, access, and care-seeking attitudes—means that SAMHI may capture unmet need differently across areas [20, 21, 22]. These issues do not invalidate SAMHI's use, but they constrain causal interpretation; we therefore keep policy claims modest and focus on relative area differences and patterning."

Minor
Comment: Page 3: "associations with other health outcomes e.g. smoking, drinking, physical activity…" would describe smoking, drinking and activity as health behaviours rather than outcomes.
Response: Thank you for the suggestion. We agree and made the change, the sentence in the text is now "**associations with health behaviours e.g. smoking, drinking, physical activity**…"

RESPONSE TO REVIEWER 3

We thank you for the thoughtful and detailed review. We have carefully revised the manuscript to address each point. Below, we provide a point-by-point response with proposed text changes (in quotation marks and bold). Page/section references correspond to the revised manuscript.

Comments related to Abstract:

Comment 1. The abstract is clear but it lacks some key details which may help prepare readers for the full text, in particular – how is "mental health" quantified, the model is applied to estimate the association, and the total "N" (i.e. number of MSOA).

Response: We have revised the design section of the abstract to include the total N (61,101 observations, for 9 years, for 6,789 small areas, i.e. MSOAs). We have also added the model applied and how mental health is quantified. It now reads (page 1):

"**Design** A longitudinal small area-level analysis exploring the association between a proxy for population mental health and economic prosperity across 6,789 small areas in England from 2011 to 2019 (N*T= 61,101). We apply linear regression models with fixed effects at the area level. Mental health in each area is proxied by a standardised index constructed from administrative data on the use of services related to mental health."

Comments related to Introduction:

Comment 2. Is a bidirectional relationship being proposed? The introduction notes that mental health can deteriorate due to economic factors, but also that mental health can be a causal factor of future income (via labour market exclusion). There is evidence to support this, but it may confuse readers because it is not explicitly stated that the effect could be two-way (i.e. present mental health affects future economic status, present economic status affects future mental health).

-I also suggest clarifying the temporal sequence in the stated aim – the association between mental health and future economic prosperity (at a population level).

Response: We agree that the introduction may inadvertently give the impression that we are proposing a causal, bidirectional relationship. Our intention is to acknowledge the existing literature, which shows evidence that mental health and economic circumstances can influence one another, while making clear that our study focuses only on one temporal direction: whether changes in population mental health are followed by changes in area level economic prosperity.

To avoid confusion, we have revised the Introduction and the stated study aim to:

1. Explicitly state that the broader literature supports a bidirectional relationship, but that
2. Our analysis examines only one direction of temporal association (mental health → future GDHI), and
3. We do not attempt to identify causal effects or rule out reverse causality.

We believe these revisions help clarify the scope of our contribution and avoid misinterpretation of our findings. We added the following text in the Introduction (last paragraph page 2):

"**The wider literature highlights a bidirectional relationship between economic conditions and mental health: economic circumstances can shape subsequent mental health outcomes, while mental health itself can influence economic prosperity. Adverse economic conditions can worsen mental health through income loss, unemployment and insecurity, while mental ill-health can reduce labour market participation and productivity, with area-level consequences for household income. In this study, however, we focus on one temporal sequence only—the association between population mental health and subsequent area-level economic prosperity.**"

We also make it clearer in the stated aim that the association studied is between population mental health and future economic prosperity at the area level. The stated aim is now the following (beginning of last paragraph page 2): **"This paper aims to fill the evidence gap on the association between a proxy for population mental health and future economic prosperity at the area level."**

Comments related to Methods:

Comment 3. A reporting checklist was not included with the manuscript – I suggest that STROBE would be mostly suitable, because this is an ecological observational study.

Response: We have now added a STROBE checklist, thank you for the suggestion.

Comment 4. The Data subsection gives a good overview of the key characteristics of the dataset, but not where it was sourced from. This is provided in later sections, but it may make more sense under "Data".
Response: We have now moved the information about the source of the data in the "Data" section before the 'outcome' subsection on page 3. We now have the following sentence: "**Data are publicly available and sourced from the ONS website, the Place-Based Longitudinal Data Resource (PLDR) [13] and Annual Population Survey (APS).**"

Comment 5. Exclusion criteria: Is it possible to give more information about the criteria used to exclude the two MSOA due to population and outcome values?
Response: We have added information regarding the reasons for exclusion of the City of London and Isles of Scilly in the paragraph 'Data'. We explain that this is common practice in small-area research to exclude the Isles of Scilly because it does not meet the population threshold to be a MSOA (threshold is 5,000 residential population while the Isles of Scilly has on average a population of 2,267). The reasoning to exclude the city of London comes from a GDHI which is 4.6 times the average, situating it in the 0.1% highest value of the GDHI distribution and significantly skewing the distribution. We have added the following text on page 3: "**Following common practice in small-area research, we excluded MSOAs in the City of London and the Isles of Scilly because of extreme values of the outcome, reflecting statistical instability. More precisely, the City of London because of extreme outcome values (4.6 times larger than the average of the sample) and the Isles of Scilly because of small population size, which is below the threshold for a MSOA."**

Comment 6. Outcome: There are a few elements which make it difficult to fully interpret this section and I Suggest considering:
-While details of how the outcome was measured in the original data are provided in C1, a brief summary in the main text would be convenient for readers.
Response: We have added a sentence defining the outcome on page 3: "**GDHI is the net amount of money that households have available, *i.e.* what they can spend or save, minus the taxes and plus any benefits received.**" and a quick summary explaining how the variable is constructed: **"To be able to compare the values between MSOAs of different size, we divide the GDHI by the population of the area."**
-"we used the log to interpret our results as changes in percentage" – suggest rephrasing as "we log-transformed prior to modelling so that coefficients could be interpreted as percentage changes", or similar, to be more precise.
Response: We have revised the sentence to the following on page 3: **"We log-transformed the variable prior to modelling to interpret the coefficients as percentage changes."**
-How the outcome is handled temporally. i.e. that it was observed annually 2011-2019, with the intention of modelling with the prior year of mental health. What was the rationale for a 1 year time lag rather than say, 2 or more years?
Response: The rationale for a one-year time lag came from a trade-off between the number of lags and the number of observations lost in the analysis because of this, as adding another year of lag leads to losing one year of data for the outcome. More precisely, if we introduce two-year lag, we do not have this information for the outcome in year 2011 and year 2012, so we lose the observations related to these years. There exist empirical tests to determine the correct number of temporal lags in time-series analyses, but these tests rely on more years of data than we currently have. We made the choice to keep the analyses simple and to keep the maximum number of observations, hence we choose only one-year lag. We have also done the analysis with adding the two-year lag, as well as the

three-year lag to check the sensitivity of our results to this choice and do not find a significant change for the one-year lag of SAMHI. We add these results in the appendix (Appendix D. Additional results, D.2 Different temporal lags results) and refer to it in the text in the method section at the beginning of the first paragraph page 5: "**We focus on the results for one period lag of SAMHI but we do explore other time lags (-2 and –3), the results are presented in Appendix D.2.**"

Comment 7. SAMHI: Is it possible to report the range of the original score, and what 1 SD unit corresponds to? This would aid with interpretation.
Response: Unfortunately the SAMHI original score before standardization is not made available by the authors who produced the data. We have however reported the score of the standardized SAMHI, hoping this would help with the interpretation. We edited the text as follows (page 6): **"The mean of the SAMHI over the period is 0.02 (Table 1), a positive value indicates a population mental health above the period mean. The range of the variable is from –6.01 to 2.07 (not shown in table). The standard deviation of 0.92 is mainly differences between MSOAs (between standard deviation is 0.78 - not shown in table), the variation within a MSOA exhibit relative persistence of the index (within standard deviation is 0.47 - not shown in table)."**

Comment 8. Model: The modelling approach is reasonable and I have no concerns regarding its application. However, I do suggest adding a few details which may help readers follow, particularly those without an econometrics background:
-Explicitly state that this is a repeated measures analysis, i.e. repeated observations of the same MSOA at different timepoints.
Response: We have added at the beginning of the section statistical analysis the indication that our data are repeated observations of the same MSOA at different timepoints. The first sentence of the paragraph on page 4 is now the following: **"Our empirical analysis relies on the longitudinal nature of the data, *i.e.* repeated observations of the same MSOA over the period 2011 to 2019."**
-The rationale for fixed effects at the area level is clear and accurate, but it may also help to state that this has the effect of accounting for non-independence from repeated measures.
Response: We have added the following sentence in the method to state this on page 4: "**In model 2, we added fixed effects at the area (MSOA) level to account for unobservable time invariant characteristics. It also accounts for non-independence of observations because of the repeated measures of the same MSOA**."
-Using the 1 year time lagged exposure has a strong rationale – to create a temporal separation which allows for a stronger interpretation of the association (compared to cross-sectional analysis). However, it would be more precise to avoid stating it accounts for reverse causality, because past GDHI can still affect future mental health and is not estimated in this analysis. Instead, it reduces reverse causality as a concern when interpreting the association of interest.
Response: We have reformulated this part of the method section. We acknowledge the issue of reverse causality, that we introduce temporal ordering to reduce it, and that it does not entirely remove reverse causality and we cannot claim causality. It now reads on page 4: "**In model 2, we added fixed effects at the area (MSOA) level to account for unobservable time invariant characteristics. The association is still likely to be biased as it might capture reverse causality, i.e. that economic prosperity causes population mental health. As we are interested in the other direction, i.e. that population mental health causes economic prosperity, we use the variable SAMHI from the previous time period to introduce a temporal ordering and reduce the concern of reverse causality. It is important to note that including lagged SAMHI values does not identify a causal effect or remove reverse causality. Economic conditions may influence mental health over multiple past periods, and SAMHI itself is relatively persistent over time. The lag specification**

therefore serves only to examine temporal ordering, not to isolate exogenous variation. As such, the models estimated associations within areas, and the directionality of the relationship cannot be definitively established. "

Comments related to Results:

Comment 9. For each table I suggest adding the N of MSOA contributing to the statistics.

Response: The number of MSOA contributing to the statistics of Table 1 are mentioned in the Table notes: **"Descriptive statistics over the period 2011-2019 of 6,789 MSOAs, excluding City of London and Isles of Scilly."**. For table 2, we added a column "MSOAs (N)" which indicates the number of MSOAs contributing to the regression. We hope this makes clear the number of MSOAs contributing to the statistics.

Comment 10. "The mean of the SAMHI over the period is 0" isn't it 0.02? The range of possible values seems to be 0-1, so I recommend not rounding it. In the sentences discussing this it may help readers to remind them that higher values reflect better area-level mental health.

Response: We have corrected the imprecision. The mean of zero for SAMHI is actually at the LSOA level, which is the construction level of the SAHMI but once we aggregate at the MSOA level, this does change the mean. The range of value of the SAMHI is actually from –6.01 to 2.07. We have added a sentence mentioning it when describing the results related to this variable on page 6 in the subsection 'Mapping regional inequalities': **"The mean of the SAMHI over the period is 0.02 (Table 1), a positive value indicates a score above the period mean. The range of the variable is from –6.01 to 2.07 (not shown in table)."**

Comment 11. Figure 1a / 2b. I suggest reporting the definitions of 1-4, e.g. are these quartiles?

Response: Thank you for the suggestion and noting that we did not define the scale of the maps. These are indeed quartiles. We made two modifications to make that clear on page 7: 1) we added 'quartiles' in the maps' titles, now respectively **" (a) Quartiles of GDHI per capita in 2019"** and **" (a) Quartiles of SAHMI in 2019"**, 2) we added the following note to each map: **"The scale represents quartiles with 1 refereeing to the first quartile and 4 to the last one."**

Comments 12-13. Table 2: There are elements of the analysis indicated in this table which were not reported in the methods and impair readability or otherwise seem unclear:

-The methods should report that there are multiple models, as currently it gives the impression of a single model, and include the rationale for each. Particularly the unlagged models, these are cross-sectional and have poorer interpretability than the longitudinal models, and it is not clear how this aligns with the aims?

Response: We have added text to make it clearer that we are running 6 models and explain that we do so to analyse the changes in the coefficients and comment on the association between the main variable of interest and the outcome. We have revised the statistical analysis section (page 4) to make it more clear: "**We ran six linear regression models with different fixed effects specifications to allow progressive comparison of the changes of the main coefficient of interest. Across, all models we assumed a log-linear association between the outcome, the log of GDHI per capita for MSOA *i* at time *t*, and the main variable of interest, SAMHI for MSOA *i* at time *t*.**"

"The first model (model 1) included year fixed effects and baseline controls. It provided us with a baseline of the association between SAMHI and the log of GDHI per capita. This model is likely biased as it does not account for unobservable time invariant characteristics at the area level which are likely to confound the association. In model 2, we added fixed effects at the area (MSOA) level to account for unobservable time invariant characteristics. The association is still likely to be biased as it

might capture the reverse causality, *i.e.* that economic prosperity causes population mental health. As we wanted to test the other direction, *i.e.* that population mental health causes economic prosperity, we introduced a time lag to avoid the simultaneity. We used the SAMHI in the previous time period $t-1$ in the other models. Model 3 had the same specifications as model 2, except that the main variable of interest is now the SAMHI in the previous time period t-1.
As we expect MSOAs within a LAD to be affected by similar shocks over time, we show the results for models where we introduced fixed effects interacting times fixed effects with LADs fixed effects to account for unobservable time varying characteristics at the LAD level (model 4). We proceeded similarly for regions (model 5) and Travel To Work Areas (TTWA), which capture labour markets (see appendix B) (model 6)."

-The N varies per model, is this due to missing data? If so I suggest adding a line to report this in the statistical analysis.
Response: The variation in N is indeed due to missing data for the controls at the LAD level, unemployment rate and percentage of population with no qualification. We have added a sentence in the statistical analysis indicating this. We also added the results of the balanced panel when we do not add these controls at the LAD level. The results remain similar.
The change in the text is the following on page 4 in the statistical analysis section: "**Due to missing values for these variables, the dataset is unbalanced. We present the results also for the balanced dataset in appendix D.1."**

-Please add the units of both the exposure and outcome to ensure the coefficient is interpreted accurately.
Response: We have now added log in front of the outcome in the title on page 8: **"Table 2: Association between log GDHI per capita and mental health"** and we have added that the exposure unit is a standard deviation by adding sd in parentheses next to SAMHI. We have also added the following in the notes of the table: "**sd: standard deviation is the SAMHI unit**."

-There is reference to "baseline controls" but this was unmentioned in the methods.
Response: Thank you for pointing out this omission. We have added the following sentences in the statistical analysis section on page 4: "**We controlled for time *t* with year fixed effects and characteristics time-varying at the MSOA level (population age groups) as well as at the LAD level (unemployment rate and percentage of population with no qualification). Due to missing values for these variables, the dataset is unbalanced. We present the results also for the balanced dataset in appendix D.1. We refer to these characteristics as baseline controls when presenting the results."**

-The statistics are reported on a 0-1 scale in the table, but then in a 0-100 (i.e., percentages scale) in the text, which may confuse readers.
Response: We have changed the text and now report the coefficients, while keeping the percentages in parentheses to ease the interpretation, notably later in the discussion. On page 7, we have the following changes:
"In our preferred specification (model (3), Table 2), a one standard deviation increase in SAMHI is associated with a 0.019 (1.9%) increase in GDHI per capita the following year."
"A one standard deviation increase of the SAMHI is associated with a positive variation of the GDHI per capita, ranging from a 0.006 (0.6%) increase to a 0.012 (1.2%) change depending on the level of time-varying unobservables accounted for."

Comments related to Discussion:

Comment 14. "The longitudinal nature of our data enabled us to control for unobservable characteristics at the area level." I think it is important to qualify this with a note that it was the MOSA fixed effects which enabled control of unobserved characteristics, and this has the assumption that the confounders are constant over time (time-invariant).

Response: Thank you for the suggestion, we agree that this was not clear and we need to make a clear link between introducing MSOA fixed effects and controlling for time-invariant unobserved characteristics. The sentence in the discussion (towards beginning on page 9) is now the following: **"The longitudinal nature of our data enabled us to introduce MSOA fixed effects in our models (models 2 to 6), which control for unobservable time-invariant characteristics at the area level."**

Comment 15. Limitations: I suggest that "reverse causality" may not be the precise issue. Typically, reverse causality would refer to a true effect of GDHI -> SAMHI contaminating the SAMHI -> GDHI association, if

-In this study, the association estimated is SAMHI_year_-1 -> GDHI_year_0, which would rule out reverse causality.

-The authors raise the concern that perhaps past GDHI could influence mental health.

E.g. "GDHI_year_-2 -> SAMHI_year_-1 -> GDHI_year_0", as well as past GDHI affecting future GDHI: "GDHI_year_-2 -> GDHI_year_-1 -> GDHI_year_0"

-However, this is more reflective of bidirectional causality and dynamic confounding. In practical terms it could mean that the association of interest is in part reflective of unmodelled income dynamics. I suggest the authors consider this and if they agree, to rephrase the present limitation, because the phrasing is a little imprecise.

Response: We agree with the fact that the issue of reverse causality described in the 'Strengths and weaknesses of the study' paragraph is not the issue causing a potential bias of our results and the issue described is related to bidirectional causality and dynamic confounding resulting from potential unmodelled income dynamics. We have now rephrased the limitation in the following way (Discussion, paragraph Strengths and weaknesses of the study, towards end of page 9) : "**However, we could not entirely rule out the possibility that the significant positive coefficient is due to bidirectional causality and dynamic confounding, *i.e.* that past GDHI per capita affect both SAHMI in the subsequent period and GDHI per capita in the subsequent period. Modelling this dynamic is out of the scope of this study, but it means that our estimates may be biased and probably an upper bound of the true effect of past mental health use on subsequent GDHI per capita.**"

VERSION 2 - REVIEW

**Reviewer**	**1**
**Name**	**Beazley, Peter**
**Affiliation**	**University of East Anglia, Clinical Psychology and Psychological Therapies**
**Date**	**22-Apr-2026**
**COI**	

Thank you for the opportunity to review a revision of this paper. I have to say I am very impressed with how the authors have worked to engage with the comments made by the reviewers, including my own comments. I can see they have worked hard to revise the paper. Having read it, I find the work to be substantially improved with only some minor editorial changes to be considered. Its relevance and value is much more clearly expressed.

1. Introduction. This seems much improved. The rationale for the study is clearer, it is more accessible for a less specialist audience, and the authors have worked hard to contextualise the potential limits of their approach from the outset. All of the changes here are welcomed. My only suggestion is consideration of a footnote or bracketed explanation for MSOA – this is the first instance of the use so abbreviation needs spelling out, but also potentially a very brief explanation that helps a non-specialist reader.

2. Method. Again, this reads much more clearly and accessibly for a non specialist audience. The description about the SAMHI and what it is (and isn't) measuring is much clearer and transparent. The comments about lag specification and causality are valued. Again, a small suggestion, but you might briefly clarify the meaning of 'deflated' (as in 'log GDHI deflated per capita') for a non-economic audience.

3. Results.

a. Table 1: some further clarification would be valued here, again with a non-specialist reader in mind. Under 'controls MSOA level' please consider clarifying what these represent in the table (I presume population by age structure noted page 5, line 13). And what is the unit for 'unemployment rate' (I presume %?)

b. Line 52, sentence starting 'The standard deviation of 0.92…' needs some clarification.

4. Discussion. Much improved given the responses to the various reviewer comments.

Figures:

I presume Page 14 is figure 1, Page 15 is figure 2, Page 16 is figure 3…. Etc.

Re: Figure 2 (GDHI by time graph) would this be clearer if Y scale was capped at £35k or £40k?

Re: Figure 3 (SAMHI map) – are 'worst mental health' and 'best mental health' the right anchors here (again just going back to the 'what does the SAMHI represent)

Reviewer 2

Name McManus, Sally

Affiliation City University of London

Date 28-Mar-2026

COI

Thanks for your full consideration of all my suggestions.

VERSION 2 - AUTHOR RESPONSE

RESPONSE TO REVIEWER 1

We would like to thank the reviewer for their helpful remarks, as well as for the appreciation shown for our responses to their comments on the initial version of the manuscript. We explain below how we address the remaining comments on the latest version of the manuscript.

Comment 1. Introduction: My only suggestion is consideration of a footnote or bracketed explanation for MSOA – this is the first instance of the use so abbreviation needs spelling out, but also potentially a very brief explanation that helps a non-specialist reader.
Answer: We agree that the MSOA abbreviation needs spelling out as it is the first instance it is used. We emphasize that MSOA-level data are geographical data. The last sentence of the introduction, at the beginning of page 3 is now:
"***By using annual geographical data at the Middle-layer Super Output Area (MSOA)-level data over nine years, we contribute new empirical evidence on temporal associations across local areas in England, while recognising that the data do not support causal inference."***
To help a non-specialist reader, we added an explanation regarding MSOA in the paragraph 'Methods-Data', which comes after the introduction:
"***MSOA is a statistical geography level defined by the Office for National Statistics containing between 2,000 and 6,000 households.*** "

Comment 2. Again, a small suggestion, but you might briefly clarify the meaning of 'deflated' (as in 'log GDHI deflated per capita') for a non-economic audience.
Answer: We added two sentences on page 3, in the paragraph 'outcome' to explain briefly what we mean by 'deflated' and why this is relevant: ***"To account for the fact that pounds in different years are not directly comparable due to differences in prices, we remove the effect of the inflation by applying the gross domestic product implicit price deflator to GDHI. We work with GDHI per capita measured in 2022 prices, also called deflated GDHI per capita in the remainder of the text."***

3. Results.
Comment 3a. Table 1: some further clarification would be valued here, again with a non-specialist reader in mind. Under 'controls MSOA level' please consider clarifying what these represent in the table (I presume population by age structure noted page 5, line 13). And what is the unit for 'unemployment rate' (I presume %?)
Answer: In Table 1, 'controls MSOA level' corresponds to control/confounder variables we have at the MSOA level, and is limited to population age structure, i.e. the variables in the lines from 'Population aged 15-29' to 'Population aged 85 and more'. To make it clearer, we renamed the sub-title **"Control variables available at MSOA level (population by age structure)".** Similarly, we renamed 'Controls LAD level' to **"Control variables available at LAD level".**
A percentage sign has been added after unemployment rate in the table to specify the unit.

Comment 3b. Line 52, sentence starting 'The standard deviation of 0.92…' needs some clarification.
Answer: We have extended this sentence to make it clearer. It is now: "***The standard deviation of 0.92 which captures how dispersed the data are from the mean is mainly due to differences between MSOAs (between standard deviation is 0.78 - not shown in table), the variation within a MSOA, i.e. dispersion in the values for the different observations of one MSOA, exhibits relative persistence of the index (within standard deviation is 0.47 - not shown in table).***"

Figures:
Comment Re: Figure 2 (GDHI by time graph) would this be clearer if Y scale was capped at £35k or £40k?
Answer: We have rescaled the graph and the Y scale is now capped at £35k.

Comment Re: Figure 3 (SAMHI map) – are 'worst mental health' and 'best mental health' the right anchors here (again just going back to the 'what does the SAMHI represent)
Answer: Thank you for spotting this. We have changed the labelling of the scale of the map to 'lowest SAMHI' and 'highest SAMHI'
